# Phytocompounds as potential inhibitors of mycobacterial multidrug efflux pump Rv1258c: an in silico approach

**DOI:** 10.1186/s13568-024-01673-9

**Published:** 2024-02-15

**Authors:** Santasree Sarma Biswas, Jayanti Datta Roy

**Affiliations:** 1 Department of Microbiology, Assam Don Bosco University, Tapesia Gardens, Sonapur, Assam 782402 India; 2 Department of Biosciences, Assam Don Bosco University, Tapesia Gardens, Sonapur, Assam 782402 India

**Keywords:** Tuberculosis, Multidrug resistant, Efflux pump, Rv1258c, In silico docking

## Abstract

The number of infections and deaths caused by multidrug resistant (MDR) tuberculosis is increasing globally. One of the efflux pumps, that makes *Mycobacterium tuberculosis* resistant to a number of antibiotics and results in unfavourable treatment results is Tap or Rv1258c. In our study, we tried to utilize a rational drug design technique using in silico approach to look for an efficient and secure efflux pump inhibitor (EPI) against Rv1258c. The structure of Rv1258c was built using the homology modeling tool MODELLER 9.24. 210 phytocompounds were used for blind and site-specific ligand docking against the modelled structure of Rv1258c using AutoDock Vina software. The best docked plant compounds were further analysed for druglikeness and toxicity. In addition to having excellent docking scores, two plant compounds—ellagic acid and baicalein—also exhibited highly desirable drug-like qualities. These substances outperform more well-known EPIs like piperine and verapamil in terms of effectiveness. This data shows that these two compounds might be further investigated for their potential as Rv1258c inhibitors.

**Supplementary Information:**

The online version contains supplementary material available at 10.1186/s13568-024-01673-9.

## Key points

- Rv1258c is an important efflux pump which provides multidrug resistance in mycobacteria.
- The structure of the pump was prepared using homology modeling and docking studies were conducted against a number of phytocompounds looking for possible efflux pump inhibitors (EPIs).
- Based on in silico studies, two phytocompounds ellagic acid and baicalein show desirable properties of being an efficient EPI.

**Supplementary Information:**

The online version contains supplementary material available at 10.1186/s13568-024-01673-9.

## Introduction

Tuberculosis, caused by *Mycobacterium tuberculosis*, is one of the most perilous infectious diseases in humans. Over the past few decades, there have been effective control methods implemented globally that have reduced the disease's fatality rate. However, there is still a long way to go before the condition can be cured. In addition, the COVID-19 pandemic has had a devastating impact on the treatment and management of tuberculosis in recent years due to the global disruption of the healthcare system. 10.6 million people were infected overall in 2021, and 1.6 million fatalities were recorded (Global Tuberculosis Report 2022). The rise of drug-resistant tuberculosis is one of the main reasons for the illness's severity and death. Multidrug resistant tuberculosis (MDR TB) cases, which are resistant to multiple drugs, have also climbed in 2021. Mycobacteria develop drug resistance through a variety of processes, including their peculiar cell wall structure, drug inactivation or modification, drug receptor inactivation, and efflux pumps.

In *Mycobacterium tuberculosis*, the main mechanism that renders them drug resistant is mutation in the target genes. However, in certain clinical isolates that were resistant to drugs, no gene mutation was found; rather, drug efflux mechanisms were used in these instances to confer drug resistance. Due to the fact that they export a wide range of diverse antibiotics, these efflux pumps are also known as multidrug-resistant (MDR) efflux pumps. (Neiderweis 2003). Since these pumps are crucial for mycobacterial drug resistance, inhibiting them with efflux pump inhibitors (EPIs) can increase the effectiveness of antibiotics against the bacteria and turn them from drug-resistant to drug-sensitive phenotypes. These compounds can be combined with current anti TB drugs, thus restoring the activity of these standard drugs. These pumps can bind to the antibiotics and block them, thus, preventing the binding of antibiotics to the pumps (Sharma et al. 2019). Hence, research into efflux pump inhibitors is crucial, leading to the development of a strategy called the EPI strategy (Kapp et al. 2018).

Despite the fact that mycobacteria include a vast number of efflux pumps, some are crucial to the survival of the bacteria as well as drug ejection. One of the important efflux pumps is Rv1258c or Tap efflux pump due to its capacity to resist the antibacterial efficacy of rifampicin, amikacin, spectinomycin, gentomycin, isoniazid, ethambutol, and pyrazinamid and some second line fluoroquinolones (Cloete et al. 2018). Due to its role in bacterial proliferation and drug resistance, this efflux pump has recently been receiving a lot of attention and has been found to be active in susceptible as well as drug resistant bacteria. (Cloete et al. 2018). It is a pump belonging to the major facilitator superfamily of transporters. Mycobacteria have been shown to have high treatment resistance due to the gene and certain of its mutations (Liu et al. 2019). Furthermore, Tap gene expression is increased in response to drugs such as ofloxacin and rifampicin, and deletion of this gene resulted in a reduction in microbe growth in culture media (Jia et al. 2022).

Due to its critical involvement in drug efflux and bacterial development, this efflux pump can serve as a key pharmacological target for the treatment of drug resistant tuberculosis, and research into neutralising the efflux pump's effect is essential. Several studies on the inhibition of Tap or Rv1258c, as well as its structure prediction, have been conducted. The structure of Tap has been predicted in several studies by using various homology modelling tools (Sharma et al. 2010; Scaini et al. 2019). Certain synthetic compounds that can function as possible EPIs were reported using a bioinformatics technique like verapamil (Cloete et al. 2018). Using in silico techniques, it was also shown that a synthetic tetrahydropyridine, NUNL02, which is a recognised EPI in other bacteria, is effective against Tap (Sciani et al. 2019). However, synthetic compounds may possess additional side effects; hence, plant based compound such as piperine was identified as potential EPIs against Rv1258c. However, due to specific drawbacks, none of them have been approved for clinical use (Sharma et al. 2010; Cloete et al. 2018). Verapamil has numerous adverse effects, including nausea, vomiting, fatigue, hypotension, and others (Singh and Eldrol 1978). Additionally, even though piperine and rifampicin have been proven to work in concert, the possibility of piperine's interactions with other medications has hindered its commercial usage (Cloete 2018). Recently, a drug repurposing study was carried out to look for other medications that might be used as Rv1258c EPIs. (Dwivedi et al. 2022). However, there are a lot of other compounds whose studies need to be carried out that may be used as EPIs against Rv1258c and may possess far fewer side effects as compared to the reported compounds.

Hence, the present work attempts to identify new EPIs of Rv1258c using the bioinformatics approach (Browne et al. 2022, Jayaram et al. 2012). Plants are the key source of natural chemicals that might work well as EPIs and may have far fewer toxic side effects as compared to synthetic ones. India has a long history of the application of herbs in the traditional medicine systems such as Unani, Ayurveda, and Siddha (Biswas et al. 2021). Many communities of India are still vastly dependent on plants for healing diseases like TB. Some examples include the root of *Calpurnia aurea*, the seed of *Ocimum basilisum*, seeds of *Piper nigrum* (Mangwani et al. 2020; Sharma and Yadav 2017)*.* However, there is a dearth of laboratory testing and computational methods for the scientific confirmation of the plants or their compounds. Keeping this in mind, the present study targeted 210 compounds for screening against the predicted structure of Rv1258c.

## Materials and methods

### Homology modeling

For initial data, preliminary homology modelling was carried out by an online database tool, SWISS MODEL. However, in order to make the structure ready for molecular docking and proper validation, homology modeling was carried out using MODELLER 9.24 (Eswar et al. 2006; Webb and Sali 2016).

The modeling experiments were conducted in the following two steps.

### Template selection and model building

The protein sequence of Rv1258c was retrieved from the Kyoto Encyclopedia of Genes and Genomes (KEGG) database (Aoki and Kanehisa 2005). Then the sequence in FASTA format was subjected to a BLAST (Basic Local Alignment Search Tool) search available on the NCBI (National Centre for Biotechnology Information) website, which looks for close homologs. BLAST did not return any results, meaning that no close homolog of the sequence is present in the database. Next, we subjected the structure to the PSIPRED webserver (http://bioinf.cs.ucl.ac.uk/psipred/), and the GeneTHREADER option was selected, which detects proteins of the same superfamily and hence, distant homologs of protein sequences (Jones 1999; McGuffin and Jones 2003). Simultaneously, we have also used MODELLER software for homology modelling of the protein. The sequence was given as input to the profile.build command in MODELLER to search for templates. MODELLER also returned only one result, i.e., 1pw4. Therefore, we considered 1pw4, which is a glycerol 3-phosphate transporter, as our template, and this template was used for model building and further analysis.

### Model refinement and validation

This model was refined further using the 3Drefine web server, which iteratively refines a model for five times using the i3Drefine algorithm (Bhattacharya et al. 2016).The model with the minimum energy was selected for further validation. Model validation was done using the Procheck web server, and the Ramachandran plot was used, which shows the energetically favourable amino acids of the protein in a graph (Laswoski et al. 1993; Laswoski et al. 1996). The ProSa web server was used to look for the ProSa Z score to check the overall quality of the model (Wiederstein and Sippl 2007).The Z score is a general indicator of model quality that measures the structure's total energy's deviation from an energy distribution derived from random conformations.

### Molecular docking

#### Preparation of the target protein

The modeled structure of Rv1258c was used as our receptor. The protein was optimised for docking using the Dockprep tool of UCSF Chimera. Hydrogen atoms and Gasteiger charges were added. The net charge was zero, and our receptor was saved in pdb format. The receptor’s energy was also minimized using UCSF Chimera software. The molecular dynamics simulation of the protein was also carried out using the MDWeb server (Hospital et al. 2012) and this structure was used for further analysis.

#### Ligands selection and preparation

For our study, 210 different plant compounds and one established efflux pump inhibitor, piperine, were selected and employed as ligands based on traditional plants as mentioned in the supplementary file (Additional file 1: Table S1). The structures of these proteins were retrieved from the Pubchem chemical library (https://pubchem.ncbi.nlm.nih.gov/) in sdf format. Using Open Babel software, the sdf format was converted to the pdb format (O' Boyle et al. 2011). The program UCSF Chimera 1.15 was used to optimise the ligands.

#### Energy minimization

To reduce their energy, the tiny molecules (ligands) were sent into the Chimera software. 5000 rounds of the conjugate gradient and 5000 cycles of the steepest descent were used to reduce the energy. The structures were minimized, after which protonation of the ligands was carried out, Gasteiger charges were added, and the files were saved in pdb format (Wang et al. 2006). The ligands were given Gasteiger-Hückel charges and AMBER ff14SB during the minimization phase (force fields). These optimised ligands were employed in the molecular docking studies.

#### Molecular docking studies

Molecular docking studies were carried out between our target protein Rv1258c and 210 ligands to explore potential EPIs from these phytocompounds. Molecular docking was carried out using AutoDock Vina (http://vina.scripps.edu) run from UCSF Chimera 1.15 (Butt et al. 2020; Pettersen et al. 2004). In order to ascertain the efficacy of the phytocompounds, both blind and site-specific docking were performed. Docking experiments were performed in triplicate for both types of docking (site-specific and blind).

#### Blind docking

For performing blind docking, the grid box was placed at the centre of the protein, and the x, y, and z coordinates were extended so as to cover the entire protein. The AutoDock Vina was run by Chimera 1.15 to find the binding affinities of the ligand to the receptor. The number of binding modes was 9. The exhaustiveness of the search was 8. The conformations were ranked according to their binding affinities. Verapamil and piperine, as previous effux pump inhibitors were docked against the efflux pump and their docking scores are taken as control.

#### Site-specific docking

Identification of probable active sites is a key issue for performing virtual screening of compounds. In this paper, to perform site-specific docking, the active site of Rv1258c was predicted using the fpocket web server (Le Guilloux et al. 2009), which detects possible cavities in the protein where small molecules can bind. For our work, we had uploaded our target protein in the fpocket web server. It returned a list of pockets ranked by their likelihood of binding a small molecule. The first pocket has the highest possibility of binding to ligands. So we selected it as our active site to perform site-specific docking. Site-specific docking was performed using a similar protocol as in blind docking, but the grid box size was fixed at 40*40*40, and the x, y, and z coordinates were 49.378, 10.375, and − 34.754, as per the active site of the protein.

#### Interaction studies through LigPlot + v.2.2.5

The plant compounds with the lowest docking scores were further evaluated in LigPlot + 2.2.5 (Laswoski and Swindell 2011). The amino acids of the protein play a crucial role in binding with the ligands. The number and types of bonds can be visualised in 2D using this software. This software can be used to visualise protein ligand complexes' hydrogen bonds and hydrophobic interactions. The creation of hydrogen bonds and hydrophobic interactions plays a crucial role in protein–ligand interactions and improves the stability of the complex. Hydrophobic interactions play a crucial role in protein–ligand interactions and improves the stability of the complex.

#### Compound screening for drug likeliness and toxicity

The top 10 compounds from blind and site-specific docking with the lowest docking score were subjected to drug likeliness and the study of ADMET properties. For a compound to be a drug, it should have some basic properties. It should not have more than 5 H bond donors, it should not have more than 10 H bond acceptors, its molecular mass should be less than 500 dalton, partition coefficient should not be greater than 5. This is called the Lipinski Rule of 5. The canonical smiles of the best ten ligands were downloaded from the Pubchem database, and they were subjected to study to determine whether these compounds follow the Lipinski rule, using five software programs, namely, Molinspiration, pkCSM, the Lipinski filters server, DruLiTo, and SwissADME (Pires et al. 2015; Umar et al. 2021; Diana et al. 2017). Following that, the compounds' ADMET properties were investigated. ADMET stands for adsorption, distribution, metabolism, excretion, and toxicity. To assess ADMET features such as P-gp substrate and inhibitor, BBB (Blood Brain Barrier), CNS (Central Nervous System), Caco-2 permeable, and toxicology parameters, the compounds' canonical smiles were uploaded to pkCSM, a publicly reachable online web server, ProTox-II server, AdmetSAR server, and ADMETlab 2.0. (Banerjee et al. 2018; Cheng et al. 2012; Motwalli et al. 2021; Dong et al. 2018). The bioavailability of a compound as a drug is also an important research area and is based on its ADME properties. The bioavailability scores of these compounds were determined using the SwissADME and admetSAR 2.0 tools. Further, the LD50 (rat) of these compounds and their toxicity class were also determined using the ProTox II web server, and these are depicted in table(Table 5).These are freely available web servers that are used to predict the ADMET properties of compounds in silico.

## Results

Rv1258c is a significant mycobacterial efflux pump that contributes to multidrug resistance by conferring resistance to numerous antibiotics. As a result, finding a potent EPI against this pump is a crucial area of research. Therefore, the present study aims to look for potent EPIs from plant sources, as these have a number of benefits as compared to synthetic sources. For the study, we retrieved the amino acid sequence of Rv1258c from KEGG having accession number T30239:34247, and it was seen that the protein consists of 419 amino acids. Initially, a BLAST search was performed, which indicated that no close homolog of the protein of interest was available. Consequently, the distant homologs algorithm was employed for predicting the structure using the PSIPRED web server. The search resulted in six templates based on a significant p value (less than 0.001), out of which a glycerol 3-phosphate transporter (1pw4) from *E.coli* was ranked at the top with a score of 331 (p = 1*10^–8^). Templates were also searched using the MODELLER 9.24 web server using the profile.buildcommand, which yielded the same outcome, suggesting 1pw4 as the template. The results were found to correlate with the structure prediction results of Cloete et al. 2018. Further, the model was built up using using Align2D command of Modeller 9.24, which yielded 10 models, and the model with the lowest DOPE (Discrete Optimized Protein Energy) score was selected as our model. A sample of native protein structures was used to calculate the DOPE, or atomic distance-dependent statistical potential. Its sole foundation is the probability theory. The joint probability density function (pdf) of the atomic Cartesian coordinates' negative logarithm is a statistical potential. As a result, the DOPE method is a statistical method for evaluating built structures and determining the most accurate structure. From a group of models created by MODELLER 9.24, the best structure can be chosen using the DOPE model score. A model is better if its DOPE score is lower. The DOPE scores along with the model are shown in table (Additional file 1: Table S2). The 9th model has the lowest DOPE score and hence, selected as the best model for the molecular docking.

This model was further refined using the 3Drefine web server, and the refined model with the lowest energy is selected for further analysis. The refined model was analysed by studying the Ramachandran plot as shown (Additional file 1: Fig. S1a) which showed that 89.1% of the residues fall in the allowed region, with 2 residues falling in the disallowed region, and a ProSa Z score of − 4.61 was obtained (Additional file 1: Fig. S1b). The combination of the backbone dihedral angles is statistically represented in the Ramachandran plot. To further analyse the predicted structure, the templates were superimposed, which showed less deviation from the folds predicted (Additional file 1: Fig. S2).

After the model was created and validated, molecular docking was carried out. In the present study, blind and site-specific docking, both approaches were adopted. Blind docking involves attaching a ligand to the entire surface of a protein without any knowledge of the target pocket. Blind docking necessitates numerous trials/runs and energy calculations prior to identifying a favourable protein–ligand complex pose. In site-specific docking, locus for ligand to bind is predicted using the f pocket web server and based on the results the grid box is designed as per the x, y and z axis values given.

In the present study, for the site-specific docking, the active site for the ligand to bind was analysed by using f pocket server. The active site of the protein and grid box around the protein is shown in figures in the supplementary file (Additional file 1: Figs. S3 and S4).

For molecular docking,AutoDock Vina software was used for both blind and site-specific docking experiments for the 210 compounds from various medicinal plants selected based on the traditional and scientific reports that they work against tuberculosis. It was found that 10 out of 210 phytocompounds showed low binding energy. These 10 ligands with their docking scores, along with the standard error of the dataset, are depicted (Table 1). The ligands N-transferuroyl-4’-O-methyldopamine, ellagic acid, abyssinone II, mollic acid glucoside, glabridine, chrysoeriol, naringenin, luteolin, isoliquiritigenin, and baicalein had blind docking scores of −9.1, −9.6, −9.6, −9.2, −9, −8.6, −8.5, −8.4, −7.9 and −9 respectively. Similarly, for site-specific docking, these compounds exhibited docking scores of −8.7, −8.6, −8.6, −8.5, −8.5, −8.5, −8.4, −8.4, −8.4, −8.3 respectively. The docking experiments were performed in triplicate, and the standard error was found to be negligible in all the sets of experiments. In the study, it was found that in blind docking, the binding energy of piperine was −9.3. Blind docking scores of −9.2 and −8.5 for piperine and verapamil, respectively, site-specific docking of verapamil and piperine showed docking scores of −5.6 and −9.2, respectively, in the present study.

**Table 1:** Docking scores along with standard error of the 10 ligands

Sl. No	Ligand	Blind docking	Standard error	Site-specific docking	Standard error
1	N-transferuroyl-4’-O-methyldopamine	− 9.1	0.066669	− 8.7	0.110868
2	Ellagic acid	− 9.6	0	− 8.6	0
3	Abyssinone II	− 9.6	0.0334	− 8.6	0.028868
4	Mollic acid glucoside	− 9.2	0.057735	− 8.5	0.028868
5	Glabridine	− 9	0.533349	− 8.5	0.46188
6	Chrysoeriol	− 8.6	0	− 8.5	0
7	Naringenin	− 8.5	0.03334	− 8.4	0.028868
8	Luteolin	− 8.4	0	− 8.4	0
9	Isoliquiritigenin	− 7.9	0.100003	− 8.4	0.086603
10	Baicalein	− 9	0.033334	− 8.3	0.028868
11	Piperine	− 9.2	0.11547	− 9.2	0.06669
12	Verapamil	− 8.5	0.128023	− 5.6	0.425584

The ten docked complexes were subjected for interaction studies in the LigPlot + v. 2.2.5 software between the ligands and Rv1258c and these are depicted in figures (Additional file 1: Fig. S5ai–tii) in the supplementary file. From the figures, we see that the ligands and Rv1258c interact mainly by hydrogen bonding and hydrophobic interactions. Blind docking analysis of N-transferuroyl-4'-O-methyldopamine reveals that the ligand and macromolecule interact via two H bonds: one with Thr51 bond length 3.04 and one with Pro361, bond length 2.81. For site-specific docking with the same compound, we find 2 H bonds- 1 H bond with Ala301, bond length 2.70 Å, 1 H bond with Ser244 bond length 3.26 Å. In the case of ellagic acid, for blind docking, we find 3 H bonds, 1 H bond with Ala48 bond length 3.14 Å, 1 H bond with Leu364 bond length 2.70 Å, 1 H bond with Ser244 bond length 3.00 Å. For site-specific docking, we find 3 H bonds- 1 H bond with Tyr146 bond length 3.16 Å, 2 H bonds with Asn151 bond length 2.80 Å each. Blind docking yields two H bonds for Abyssinone II: one with Gly 117 (bond length 2.70) and one with Asp23 (bond length 2.93).In case of site-specific docking we not see any H bonds, but hydrophobic interactions are responsible for docking. The residues involved in hydrophobic interactions are Val245, he246, Phe330, Ala 306, Leu311, Lys249, Gly368, Ser45, Tyr250, Ser45, Ala367, Val245, Leu343, Phe247, Ser244. The ligand, mollic acid glucoside, has 5 H bonds involved in the blind docking interaction.These are two H bonds with Asn221 with bond lengths of 3.28 and 2.21, respectively, two H bonds with Val224 with bond lengths of 3.02 and 2.70, and one H bond with Leu222 with bond length of 2.64.In the case of site-specific docking, we see 3 H bonds- H bond with Ala319 bond length 3.10, 1 H bond with Leu317 bond length 3.05, 1 H bond with Ala266, bond length 3.01 Å. In the case of glabridine for blind docking, we have found 2 H bonds with Arg124 with bond length 2.82 Å and 3.35 Å respectively. For site-specific docking, we find only 1 H bond at Gly368 bond length 2.93 Å. In the case of chrysoeriol, for blind docking, we see 4 H bonds- 1 H bond with Arg134, bond length 3.24 Å, 3 H bonds with Arg124 with length 3.10 Å, 2.82 Å, 2.99 Å respectively. For site-specific docking with the same compound, we find 2 H bonds- 1 H bond with Thr290, bond length 2.91 Å, 1 H bond with Asn151, bond length 2.92 Å. In the case of naringenin, for blind docking, we see 4 H bonds Bond lengths for three H bonds with Asp 79 are 2.72, 2.91, and 3.31, respectively, and one H bond with Ser 26 is 2.75.In the case of luteolin, for blind docking, we find 2 H bonds- 1 H bond with Thr290, bond length 2.96 Å, 1 H bond with Asn151 bond length 3.01. For site-specific docking, we find 3 H bonds- 1 H bond with Thr290 bond length 2.92 Å, 2 H bonds with Asn151 bond length 2.92 Å and 3.00 Å respectively. For isoliquiritigenin, in blind docking we find 2 H bonds- 2 H bonds with Gln329 with bond length 3.02 Å and 3.22 Å respectively. For site-specific docking, we do not find any H bonds. Only hydrophobic interactions are present and the residues involved are Val245, Phe330, Leu383, Lys249, Thr379, Thr371, Tyr250, Ala306, Phe245, Val245, Phe380. In the case of baicalein, for blind docking, we find 4 H bonds- 1 H bond with Leu246 bond length 2.71 Å, 2 H bonds with Ser244 bond length 2.78 Å, 3.06 Å, 1 H bond with Leu364 bond length 2.71 Å respectively. For site-specific docking, we find 3 H bonds- 1 H bond with Tyr146 bond length 2.88 Å, 2 H bonds with Asn151 with bond length 2.98 Å, 2.94 Å respectively.

Additionally, 10 compounds selected based on the docking results were subjected to drug likeliness studies using different web servers, namely Molinspiration, pkCSM, the Lipinski filters server, DruLiTo, and SwissADME and the results are depicted in table (Table 2). The ADMET properties of these compounds were also assessed using various web servers, namely, pkCSM, ProTox-II, admetSAR 2.0, and admetLab 2.0. and the results are compiled (Table 3). From the drug-likeness studies, we find that except for mollic acid glucoside, all the other phytocompounds follow all the Lipinski Rule of 5. Mollic acid glucoside has two violations: a higher molecular weight and a larger number of H bond donors. ADMET analysis showed that compounds showed acceptable ADMET properties, and the results were consistent across four different web servers. However, one of the compounds, glabridine, exhibits AMES toxicity using the admetSAR 2.0 tool and is carcinogenic using the ADMETlab 2.0 tool. Naringenin, isoliquiritigenin were also carcinogenic when using the same tool. Luteolin and isoliquiritigenin are also seen to be positive for AMES toxicity using ADMETlab 2.0. Luteolin is also seen to be positive for mutagenicity and carcinogenicity using ProTox-II software. Using ADMETlab 2.0, we can see that our control piperine is toxic and carcinogenic to AMES.

**Table 2:** The drug-likeliness properties of the ten phytocompounds (This table should be in line no. 280)

S. No	LIGANDS	Molinspiration software					pkCSM server					Lipinski Filters server					DruLiTo software			SwissADME server		
		*Mol. weight*	*LogP*	*H-bond acceptor*	*H-bond donor*	*N violations*	*Mol. weight*	*LogP*	*H-bond acceptor*	*H-bond donor*	*N violations*	*Mol. weight*	*LogP*	*H-bond acceptor*	*H-bond donor*	*N violations*	*Lipinski rule*	*Veber rule*	*Ghose rule*	*Lipinski rule*	*Veber rule*	*Ghose rule*
1	N-transferuroyl-4’-O-methyldopamine	343.38	2.25	6	3	0	343.379	2.48	3	5	0	343	2.487099	6	3	0	Yes	Yes	Yes	Yes	Yes	Yes
2	Ellagic acid	302.19	0.94	8	4	0	302.194	1.318	8	4	0	302	1.241199	8	4	0	Yes	Yes	Yes	Yes	No, 1 violation	Yes
																					TPSA > 140	
3	Abyssinone II	324.38	4.45	4	2	0	324.376	4.313	4	2	0	324	4.3129	4	2	0	Yes	Yes	Yes	Yes	Yes	Yes
4	Mollic acid glucoside	634.85	4.32	9	6	2	635.841	4.0286	8	6	2	634	4.0285	9	6	2	Yes	Yes	Yes	No	No	No
5	Glabridine	324.38	4.2	4	2	0	324.376	4.007	4	2	0	324	4.0006	4	2	1	Yes	Yes	Yes	Yes	Yes	Yes
6	Chrysoeriol	300.27	2.28	6	3	0	300.27	2.5854	6	3	0	300	2.4281	6	3	0	Yes	Yes	Yes	Yes	Yes	Yes
7	Naringenin	272.26	2.12	5	3	0	272.256	2.5099	5	3	0	272	2.5098	5	3	0	Yes	Yes	Yes	Yes	Yes	Yes
8	Luteolin	286.24	1.97	6	4	0	286.239	2.2824	6	4	0	286	2.1251	6	4	0	Yes	Yes	Yes	Yes	Yes	Yes
9	Isoliquiritigenin	256.26	2.77	4	3	0	256.257	2.6995	4	3	0	256	2.6994	4	3	0	Yes	Yes	Yes	Yes	Yes	Yes
10	Baicalein	270.24	2.68	5	3	0	270.24	2.5768	5	3	0	270	2.4195	5	3	0	Yes	Yes	Yes	Yes	Yes	Yes

**Table 3:** ADMET properties of the ten phytocompounds (This table should be in line 281 as mentioned in the text)

Ligand	pkCSM Server												
	Absorption			Distribution		Metabolism				Excretion	Toxicity		
	P-gp substrate	P-gp 1 inhibitor	P-gp 2 inhibitor	BBB permeability (> 0.3)	CNS permeability (> -2)	CYP2D6 substrate	CYP2D6 inhibitor	CYP3A4 substrate	CYP3A4 inhibitor	Renal OC2 substrate	AMES toxicity	Hepatotoxicity	Skin sensation
N-transferuroyl-4'-O-methyl dopamine	Yes	No	No	− 0.834	− 2.682	No	No	Yes	No	No	No	No	No
Ellagic acid	Yes	No	No	− 1.272	− 3.533	No	No	No	No	No	No	No	No
Mollica acid glucoside	No	No	No	− 1.04	− 3.609	No	No	Yes	No	No	No	No	No
Abyssinone II	Yes	Yes	No	0.502	− 1.854	No	No	Yes	Yes	No	Yes	No	No
Glabridine	Yes	Yes	No	0.087	− 1.8	No	No	Yes	Yes	No	No	No	No
Chrysoeriol	Yes	No	No	− 0.943	− 2.32	No	No	No	No	No	No	No	No
Naringenin	Yes	No	No	− 0.578	− 2.215	No	No	No	No	No	No	No	No
Luteolin	Yes	No	No	− 0.907	− 2.251	No	No	No	No	No	No	No	No
Isoliquiritigenin	Yes	No	No	− 0.717	− 2.205	No	No	No	No	No	No	No	No
Baicalein	Yes	No	No	− 1.061	− 2.21	No	No	No	No	No	No	No	No
Piperine	Yes	Yes	No	− 0.102	− 1.879	No	No	Yes	No	Yes	No	Yes	No
Verapamil	Yes	Yes	Yes	− 0.647	− 2.484	No	Yes	Yes	Yes	Yes	No	No	No

Ligand	ProTox-II			admetSAR 2.0		ADMETlab 2.0	
	Toxicity prediction			Toxicity prediction		Toxicity prediction	
	Mutagenicity	Cytotoxicity	Carcinogenicity	AMES toxicity	Carcinogenicity	AMES toxicity	Carcinogenicity
N-transferuroyl-4'-O-methyl dopamine	Inactive	Inactive	Inactive	Non-AMES toxic	Non-carcinogenic	Negative	Negative
Ellagic acid	Inactive	Inactive	Inactive	Non-AMES toxic	Non-carcinogenic	Negative	Negative
Mollica acid glucoside	Inactive	Inactive	Inactive	Non-AMES toxic	Non-carcinogenic	Negative	Negative
Abyssinone II	Inactive	Inactive	Inactive	Non-AMES toxic	Non-carcinogenic	Negative	Negative
Glabridine	Inactive	Inactive	Inactive	AMES toxic	Non-carcinogenic	Negative	Positive
Chrysoeriol	Inactive	Inactive	Inactive	Non-AMES toxic	Non-carcinogenic	Negative	Negative
Naringenin	Inactive	Inactive	Inactive	Non-AMES toxic	Non-carcinogenic	Negative	Positive
Luteolin	Active	Inactive	Active	Non-AMES toxic	Non-carcinogenic	Positive	Negative
Isoliquiritigenin	Inactive	Inactive	Inactive	Non-AMES toxic	Non-carcinogenic	Positive	Positive
Baicalein	Inactive	Inactive	Inactive	Non-AMES toxic	Non-carcinogenic	Negative	Negative
Piperine	Inactive	Inactive	Inactive	Non-AMES toxic	Non-carcinogenic	Positive	Positive
Verapamil	Inactive	Inactive	Inactive	Non-AMES toxic	Non-carcinogenic	Negative	Negative

**Fig. 1:**
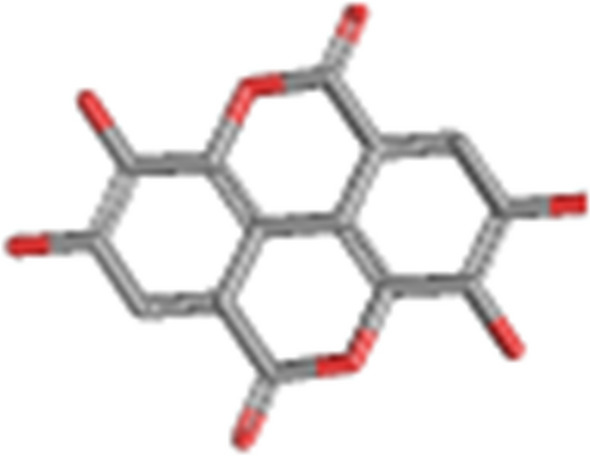
.

**Fig. 2:**
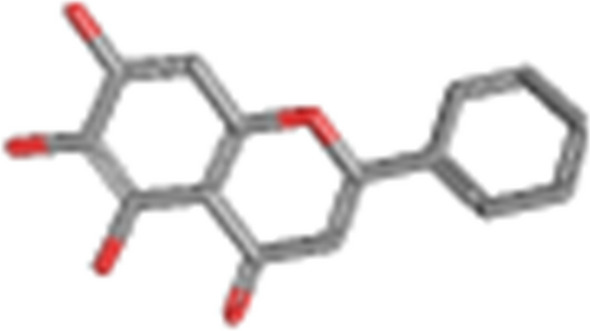
.

Using two programmes, admetSAR 2.0 and SwissADME, the bioavailability scores of the 10 compounds were assessed (Table 4). In order to evaluate acute oral toxicity, we used the LD50 test. The LD50 is the dosage of a medication required to render 50% of the test animals dead. The lower a drug's LD50 value, the more lethal it is. The substances are classified according to their toxicity, with class 1 being the most dangerous, classes 2 and 3 being moderately harmful, and classes 4 and 5 being the least toxic. None of the ten phytocompounds were found to be harmful. The ProTox-II software was used to analyse the LD50 (rat) of the 10 compounds, and the same server was also used to determine their toxicity class (Table 5). The pkCSM software showed that these substances are efficiently absorbed by the human digestive system. The oral route of drug administration is the most widely used and preferred method because it is cost-effective, non-invasive, and patient-compliant, making it a crucial factor in determining the fate of novel medications and EPIs. Using SwissADME and admetSAR 2.0 software, it is observed that the compounds ellagic acid and baicalein have high bioavailability scores. They have a bioavailability score of 0.55 in SwissADME and 67.14 and 67.1 in admetSAR 2.0 for ellagic acid and baicalein, respectively. None of the other compounds have a bioavailability score of more than 50, which is the threshold for good bioavailability according to admetSAR 2.0. Ellagic acid and baicalein both have excellent LD50 values of 2991 mg/kg and 3919 mg/kg, respectively, and are classified as having toxicity classes 4 and 5, according to the ProTox-II web server. This is much better than piperine, which has a bioavailability score of 42.86 using admetSAR 2.0 and 0.55 using SwissADME software. Additionally, it belongs to toxicity class 4 using the ProTox-II web server and has a very low LD50 of 330 mg/kg. The 3D chemical structures of ellagic acid and baicalein are depicted in Figs. 1 and 2 (Table 6).

**Table 4:** Bioavailability scores of the ten phytocompounds

Bioavailability score			
Sl. No	Name of the compound	Adsmet SAR2.0	SWISS ADME
1	N-transferuroyl-4’-O-methyldopamine	0.5	0.55
2	Ellagic acid	67.1^*^	0.55^*^
3	Abyssinone II	27.14	0.55
4	Mollic acid glucoside	17.14	0.11
5	Glabridine	37.14	0.55
6	Chrysoeriol	42.86	0.55
7	Naringenin	25.71	0.55
8	Luteolin	42.86	0.55
9	Isoliquiritigenin	47.14	0.55
10	Baicalein	67.14^*^	0.55^*^
11	Piperine	42.86	0.55
12	Verapamil	61.45	0.55

*The phytocompounds with the highest bioavailabililty scores

**Table 5:** Oral toxicity scores of the ten phytocompounds

Sl. No	Name of phytocompound	LD50 (mg/kg) (ProTox-II)	Toxicity class (ProTox-II)
1	N-transferuroyl-4’-O-methyldopamine	500	4
2	Ellagic acid	2991^*^	4
3	Mollic acid glucoside	1500	4
4	Abyssinone II	1190	4
5	Glabridine	500	4
6	Chrysoeriol	4000	5
7	Naringenin	2000	4
8	Luteolin	3919	5
9	Isoliquiritigenin	3600	5
10	Baicalein	3919^*^	5
11	Piperine	330	4
12	Verapamil	108	3

**Table 6:** Chemical structures of the top two best compounds

S No	Name of the phytocompound	Chemical structure of the phytocompounds
1	Ellagic acid	Figure 1

		Fig. : 3D chemical structure of ellagic acid. Image source:Pubchem
2	Baicalein	Figure 2

		Fig. : 3D chemical structure of baicalein. Image source:Pubchem

## Discussion

The ideal antibiotic adjuvant should have appropriate drug like characters, low toxicity and preferable bioactivity. Due to a lack of one or more of these properties, many adjuvants failed clinical trials. Drug discovery efforts have focused on finding Rv1258c system inhibitors and has also led to the identification of promising leads: piperine and verapamil have shown to work as adjuvant in *M.tuberculosis* overexpressing Rv1258cefflux pumps (Sharma et al. 2010, Cloete et al. 2018). Despite promising in vitro activity, high systemic toxicity and low bioavailability prevented both classes of compounds from being used clinically. A few synthetic molecules that might function as possible EPIs have been mentioned in several docking-based studies, although synthetic compounds almost invariably have drawbacks. Therefore, the problem of side effects and biocompatibility can be solved by the search for natural substances, particularly those derived from plant sources. Due to their high toxicity and poor pharmacokinetic characteristics, most antibiotic adjuvants were clinically ineffective (Abdel-Halim et al. 2019). The current work tries to create a trustworthy structure of Rv1258c and performs molecular docking tests on 210 plant chemicals to check for potential inhibitory activity against Rv1258c.

The availability of crystal structures is necessary for a good start to look for potential efflux pump inhibitors. But in this case the structure of Rv1258c is yet to be elucidated experimentally. Therefore, we had to depend on bioinformatics tools to predict the molecular structure of Rv1258c. The protein sequence of Rv1258c was downloaded from KEGG database and a reliable and famous software Modeller 9.24 was used to build the structure of Rv1258c. One drawback of computational modelling is the deviance of predicted models from their true, native structures as determined by experiment. To address this problem, refinement of the predicted model is necessary. Refinement of the model helps to achieve the most native like conformation of the protein. Our model was refined using 3D refine web server. The 3Drefine web server uses the optimization of an energy bonding network and an energy minimization process to refine structures. The correct conformation of interacting residues and atoms at the interface is the goal of structure refinement, which is essential for the practical application of computational protein docking models. The built structure was further validated using the Ramachandran Plot and ProSa Z score. The combination of the backbone dihedral angles is statistically represented in the Ramachandran plot. Protein structural scientists can learn more about the structure of peptides and determine which torsional angles are allowed by creating a Ramachandran plot. The total energy of the structure deviates from an energy distribution derived from random conformations, and this deviation is measured using the Z-score, which measures the overall model quality. Z-scores that fall outside the range of native protein Z-scores signify incorrect structures. Our predicted structure had a Z score of -4.61 which is not very good but is acceptable as is shown (Additional file 1: Fig. S1b). Our structure seemed to correlate with the one previously built by Cloete et al. For our study we tried to find both blind and site-specific docking scores for 210 plant compounds. Blind docking scans the entire protein target surface to find peptide ligand binding sites and modes. So in such a way the ligand binds to in any compatible locus.For site-specific docking the binding site of the protein is either found experimentally or predicted using software. For our study we found the probable binding site using fpocket web server. The binding energies of the ligands were compared with piperine and verapamil. Sharma et al. 2010 reported that piperine was a powerful inhibitor of Rv1258c by site-specific docking using the Schrodinger software and in vitro tests. Cloete et al. have also demonstrated piperine to be a potential Rv1258c inhibitor using docking experiments. In the study, it was found that in blind docking, the binding energy of piperine was − 9.3. Verapamil, a known synthetic EPI of other efflux pumps showed a binding energy of − 8.4 (Kapp et al. 2018). Based on this background, blind docking scores of −9.2 and −8.5 for piperine and verapamil, respectively, were obtained, which correlated with the findings of Cloete and coworkers. Site-specific docking of verapamil and piperine showed docking scores of −5.6 and −9.2, respectively, in the present study. Hence, piperine and verapamil were considered as controls for our study. The present study showed that the top ten compounds in terms of binding energy exhibited better and/or comparable binding energy as compared to piperine and verapamil. This indicates that all these compounds exhibit remarkable biochemical interactions at the binding site of the protein. Therefore, it may be summarised that these 10 compounds may possess the potential to bind to the efflux pump with better affinity, and hence, by competitive binding they can increase the intracellular concentration of antibiotics.

Next, to optimize the development of potential EPIs, the physico chemical properties of these compounds are necessary. Therefore, these properties of the ten compounds were carefully investigated using five available web servers. Nine out of the ten compounds followed all the Lipinski rules. After this, the critical pharmacokinetic parameters or ADMET properties were studied using four web servers. Before entering clinical trials, a drug candidate's intolerable toxicity must also be investigated. A crucial factor in drug designing is a compound's oral drug bioavailability. Because of their low bioavailability, most EPIs are not administered with drugs. We are yet to come across any research on the bioavailability and toxicity of plant chemicals in the hunt for Rv1258c's EPI. High oral bioavailability is a term used to describe when a medication can be administered orally in small doses and still reach the target and carry out therapeutic activity, reducing the risk of side effects in patients. Based on all the results, we see that most of the phytocompounds are not very toxic but two compounds—ellagic acid and baicalein exhibit the highest bioavailability scores (Table 4). According to the LD50 values also, these compounds are shown to be non-toxic, and they belong to toxicity classes 4 and 5 respectively (Table 5).

Therefore, from the 210 compounds that were screened, two phytocompounds—ellagic acid, and baicalein—show good docking scores, have excellent ADMET characteristics, and their bioavailability is superior to piperine and are non-toxic. Thus, our in silico study indicates that these two compounds have no negative effects and have the potential to be an effective efflux pump inhibitor. Therefore, based on computer-based drug design methodology, we conclude that these two compounds are possible inhibitors against Rv1258c. However, the stability of the ellagic acid and baicalein complex with pump requires molecular dynamics simulation followed by in vitro experimentation. The present study could provide leads to new class of compounds which may be used EPI against Rv1258c like efflux pumps and might cause the reversal of the antibiotic resistant condition in tuberculosis.

### Supplementary Information

**Additional file 1: Figures S1–S5 and Tables S1–S2.**

## Data Availability

The authors declare that the data supporting the finds are available in the article along with websites and in the supplementary information file.
