# Supplementary material for: Phytocompounds as potential inhibitors of mycobacterial multidrug efflux pump Rv1258c: an in silico approach
Source: AMB Express. 2024 Feb 15;14:25. doi: 10.1186/s13568-024-01673-9 (PMC10869325; doi:10.1186/s13568-024-01673-9)
Supplement: Supplementary file 1 — Additional file 1: Figures S1–S5 and Tables S1–S2. [file 13568_2024_1673_MOESM1_ESM.pdf]

Journal Name: AMB Express

Manuscript title: Phytocompounds as potential inhibitors of mycobacterial multidrug efflux pump Rv1258c: an *in silico* approach

Name of the authors: Santasree Sarma Biswas, Jayanti Datta Roy

Department of Microbiology, Assam Don Bosco University, Tapesia Gardens, Sonapur, Assam, 782402, India

Corresponding author: Jayanti Datta Roy

Department of Biosciences, Assam Don Bosco University, Tapesia Gardens, Sonapur, Assam, 782402, India
jayanti.roy@dbuniversity.ac.in
Telephone no. +919707032955

We would like to present the supplementary materials with reference to the manuscript titled above in this file.

Fig. S1a

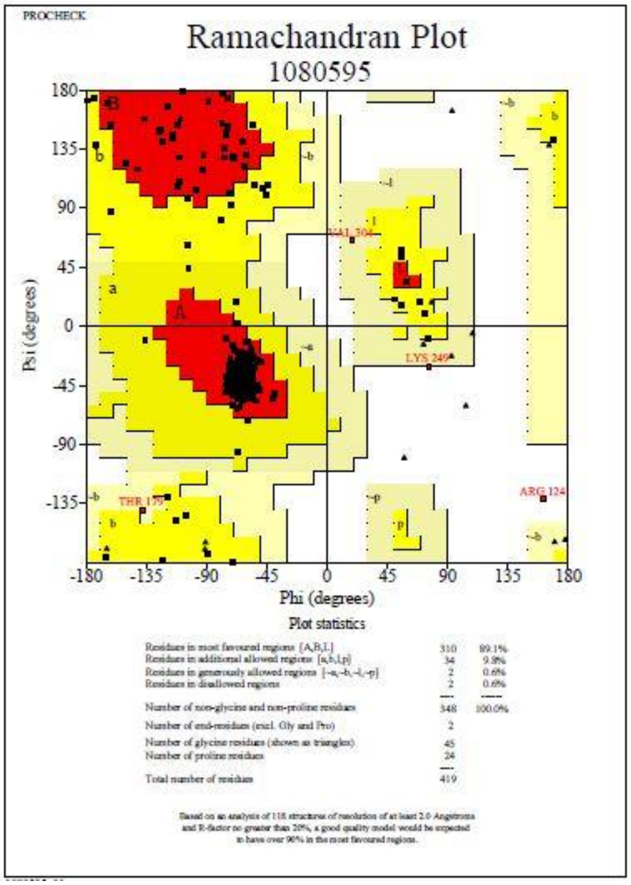

Ramachandran plot of the 9th model

Fig.S1b

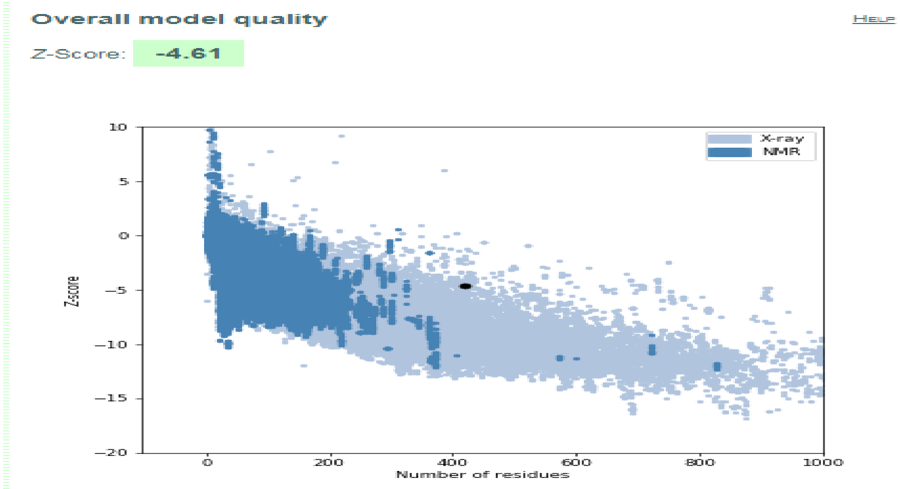

ProSa Z score of the 9th model

Fig. S2

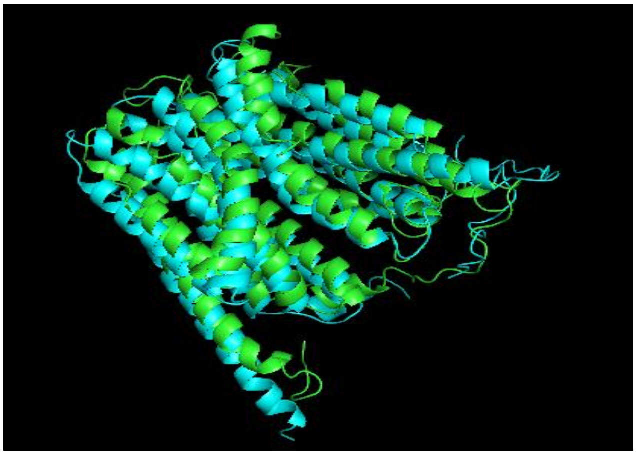

Superimposed structure of template and the model

Table S1

Sl. No.	Name of phytocompound	Name of the plants
1.	Glabridine	*Glycirrhiza glabra*
2.	Naringenin	Citrus fruit likeorange
3.	Baicalein	*Scutellaria* baicalensis
4.	Daphnoretin	*Wikstromia indica*
5.	Abyssinone II	*.Broussonetia papyrifera*
6.	Shikonin	*Lithospermum erythrorhizon*
7.	Isocolumbin	*Penianthus zenkeri*
8.	Andrograholide	*Andrographis paniculata* Nees
9.	Hesperidin	Citrus fruits like oranges. Grape fruit
10.	Catechin	*Camellia sinensis*
11.	Hesperitin(Citrus fruits)	Citrus fruits
12.	Curcumin	*Curcuma longa*
13.	Dihydroartemisinin	*Artemisia annua*
14.	Oleanolic acid	*Olea europaea*
15.	Kaempferol	*Kaempferia galanga*
16.	Pinoresinol	*Styrax sp., Forsythia suspensa, and in Forsythia koreana*
17.	Betulin	bark of birch trees
18.	Betulinic acid	birch, eucalyptus and plane trees
19.	Olealonic acid	apples, figs, cranberries
20.	Ursolic acid)	rosemary, marjoram, lavender, thyme, and organum, fruits (apple fruit peel), flowers, and berries
21.	Dihydrolycorine	*Lycoris radiata, Pancratium maritimum, and Galanthus trojanus*
22.	Pheophorbide A	Chlorophyll derivatve
23.	Nordihydroguaretic acid	*_Larrea tridentate*
24.	Epicatechin gallate	*Camellia sinensis*
25.	Chlorogenic acid	eggplants, coffee beans, carrots, kiwi, plums, potatoes etc.
26.	Diospyrine	*Diospyros lycioides*
27.	Lupeol	*Crataeva nurvala*
28.	Pheophorbide B	*Taraxacum formosanum*
29.	Tinoprin	*Tinospora cordifolia*
30.	Mirificin	*Pueraria mirifica and Pueraria lobata*
31.	Paladin	*Allium vineale, Psidium guajava*
32.	Norboldine	*Lindera umbellata, Damburneya salicifolia*
33.	Corosolic acid	*Plectranthus mollis*
34.	3,5 Dicaffeoylquinic acid	*Artemisia argyi*
35.	Methylxanthine	*Camellia sinensis*
36.	Pectoliranin	*Cirsium japonicum*
37.	6-Gingerol	*Zingiber officinale*
38.	Madecassic acid	*Siphoneugena densiflora, Centella erecta, and Centella asiatica*
39.	SkullcapflavoneII	*Lagochilus meiacanthus, Scutellaria guatemalensis*
40.	Diosmin	*Citrus sinensis*
41.	Falcarindiol	*Anthriscus nitida, Chaerophyllum aureum*
42.	Conessine	*Olarrhena floribunda, Funtumia elastica, and Holarrhena pubescens*
43.	Litseagermacrane	*Litsea verticillata*
44.	Asiatic acid	*Centella asiatica*
45.	Artemisisnin	*Microliabum polymnioides, Artemisia tenuisecta*

46.	Stigmasterol	*Xylopia aromatica, Ficus septica*
47.	Eriosemation	*Eriosema tuberosum and Lupinus luteus*
48.	Hydroxychloroquine	*Cinchona officinalis*
49.	Rutin	*Salix atrocinerea, Ficus virens*
50.	Gallotanin	*Mallotus japonicus*
51.	Plumbagin	*Plumbago zeylanica*
52.	Caffeic acid	*Coffea arabica,Punica granatum*
53.	Genistin	*Ficus septica, Dalbergia sissoo*
54.	Magnoflorine	*Zanthoxylum myriacanthum, Fumaria capreolata*
55.	Reserpine	*Rauwolfia serpentine*
56.	Berberine	European barberry, goldenseal, goldthread, Oregon grape, phellodendron, and tree turmeric
57.	Beta sitosterol	rice bran, wheat germ, corn oils, soybeans, and peanuts
58.	Grandinol	*Eucalyptus perriniana, Eucalyptus globules*
59.	Carvacrol	*Origanum vulgare, Lepidium flavum, Thymus vulgaris, Citrus aurantium bergamia*
60.	Capsaicin	Chillies, oregano, cinnamon, cilantro
61.	Bullatantriol	*Schisandra plena, Chimonanthus praecox, and Homalomenaaromatica*
62.	Beilschminola	*Beilschmiediatsangii*
63.	Thymol	*Thymus vulgaris L., different species of the genus Satureja L., Oliveriadecumbens Vent and also other plants such as Ocimumgratissimum L., Origanum L., Carum copticum L*
64.	Scopoletin	*Ficus auriculata, Haplophyllum cappadocicum*
65.	Olympicin A	*Hypericum olympicum*
66.	Syringaresinol	*Ficus septica, Pittosporum illicioides*
67.	Hyperin	*Lotus ucrainicus, Visneamocanera*
68.	Magnolin	*Geranium platyanthum, Licaria armeniaca*
69.	Eugenol	*Syzygium aromaticum*
70.	Roseside	*Elaeocarpus japonicus, Macaranga tanarius,*
71.	Palmatine	*Berberis poiretii, Coptis chinensis var. brevisepala*
72.	Vitamin E	Almonds and hazelnuts
73.	Theobromine	Cocoa beans, Tea
74.	Myristicin	*Chaerophyllum azoricum, Peperomia bracteata*
75.	Mangiferin	*Salacia chinensis, Smilax bracteata*
76.	Diacetylcurcumin	*Curcuma longa*
77.	Elemicin	*Anemopsis californica, Asarum celsum*
78.	Vanillic acid	*Ficus septica, Haplophyllum cappadocicum,*
79.	Quinic acid	*Gambleainnovans, Pterocaulon virgatum*
80.	Aloeresin	*Aloe arborescens*
81.	Ascorbic acid	Citrus fruits
82.	Ajoene	*Allium sativum*
83.	Allicin	*Allium sativum*
84.	Allyl sulphide	*Allium sativum*
85.	Deoxykaemferol(5 deoxykaemferol)	*Pterocarpus marsupium, Anthyllis vulneraria*
86.	Deoxyquercetin	*Acacia carneorum, Acacia buxifolia,*
87.	2S-sambunigrin	*Acacia lasiocalyx, Prunus spinosa*
88.	Anthraquinone	*Rheum palmatum L., Rheum tanguticum Maxim* etc.
89.	5,7 dimethoxycoumarin	*Edgeworthia chrysantha, Melicope borbonica*
90.	Apigenin	*Verbascum lychnitis, Carex fraseriana*
91.	Niacin	Peanuts, quinoa etc.
92.	Camphor	*Cinnamomum camphora*
93.	Camphene	*Xylopiaaromatica, Xylopia sericea*
94.	Limonene	Citrus fruits peels

95.	1,8-cineole	*Alpinia galanga*
96.	Geraniole	*Cinnamomum tenuipilum, Valeriana officinalis*
97.	Pinene	*Xylopia aromatica, Teucrium montanum*
98.	Lysergol	*Calonyction muricata*
99.	1′-S-1′-Acetoxychavicol acetate	*Alpinia conchigera, Apiscerana, and Alpinia galanga*
100.	Myrcene	hops, cannabis, lemongrass, verbena and bay as well as in citrus fruits and citrus juices
101.	1′-S-1′-acetoxyeugenol acetate	*Alpinia galanga*
102.	Pinocembrin	*Prunus leveilleana, Alpinia rafflesiana*
103.	Quercetin	Onions, grapes, berries, cherries, broccoli, and citrus fruits
104.	Asiaticoside	*Akebia trifoliata, Heptapleurum heptaphyllum*
105.	Dihydrocapsaicin	*Capsicum pubescens, Capsicum annuum var. annuum*
106.	Resveratrol	Grapes, wine, peanuts, and soy
107.	Ellagic acid	Berries, pomegranates, grapes, and walnuts
108.	Tannic acid	*Achillea millefolium, Calluna vulgaris*
109.	Farnesol	citronella, lemon grass, tuberose, cyclamen, rose, neroli, balsam, and musk
110.	6-paradol	*Aframomum angustifolium, Aframomum melegueta, and Zingiber officinale*
111.	8-gingerol	*Zingiber officinale*
112.	6-Dihydroparadol	*Zingiber officinale*
113.	Catechol	*Populus tremula, Aloe ferox*
114.	Piperidine	*Piper nigrum, Euglena gracilis*
115.	Biochanin A	*Dalbergia oliveri, Dalbergia sissoo*
116.	Nobiletin	*Citrus tankan, Citrus keraji*
117.	Abyssinone II	*Erythrina abyssinica and Maackia amurensis*
118.	Isorhamnetin	*Hippophaerhamnoides L. , Ginkgo biloba L.*
119.	Luteolin	*Verbascum lychnitis, Carex fraseriana, Plantago major*
120.	Taxifolin	*Salix atrocinerea, Austrocedrus chilensis*
121.	Tangeretin	Citrus fruits
122.	Wogonin	*Scutellaria likiangensis, Scutellaria amoena*
123.	Leucocyanadin	*Ixora coccinea*
124.	Procyanidin A2	*Ixora coccinea*
125.	Procyanidin B5	*Chrysophyllum albidium*
126.	Isopterocarpolone	*Pterocarpus santalinus*
127.	Furanocembranoid	*Croton oblongifolius*
128.	Eudesmin	*Machilus kurzii, Magnolia salicifolia*
129.	Cordifolin	*Tinospora cordifolia*
130.	Chrysanthemic acid	*Chrysanthemum indicum*
131.	Nepodin	*Rumex dentatus, Rumex alpinus*
132.	Bisdemethoxycurcumin	*Curcuma amada, Curcuma kwangsiensis*
133.	Methyl alpha-L-arabinopyranose	*Oryza sativa*
134.	Honokiol	*Illicium simonsii, Illicium fargesii*
135.	O-Spiroketalglucoside	*Lemna japonica*
136.	Swerilactone	*Swertia mileensis*
137.	CrotonkininA	*Croton kongensis*
138.	Crodamide O	*Desmos cochinchinensis*
139.	Iloprost	*Crotalaria prostrata*
140.	Norcepharadione B	*Houttuynia cordata, Friesodielsia velutina*
141.	Piperolactum A	*Piper auritum, Aristolochia cucurbitifolia*
142.	Cepharadione B	*Piper arborescens, Piper auritum*
143.	Terpinolene	*Xylopiaaromatica, Xylopia sericea*

144.	Beta pinene	*Nepeta nepetella, Teucrium montanum*
145.	2S naringenin	Orange, tomatoes, oregano, cherries
146.	Pilloin	*Chromolaena odorata, Alnus japonica*
147.	luteolin 7-methyl ether(7 o methyl luteolin)	*Verbascum lychnitis, Salvia hypoleuca*
150.	Chrysoeriol	*Haplophyllum ramosissimum, Myoporum tenuifolium*
151.	5-hydroxy-7,4′-dimethoxyflavone	*Haplopappus sonorensis*
152.	3-epi-betulinic acid	*Microtropis borneensis, Hypericum laricifolium*
153.	4-epi-fridelin	*Salacia chinensis, Syzygium formosanum*
154.	Baicalin	*Scutellaria amoena, Thalictrum baicalense*
155.	Rhamnocitrin	*Ageratina altissima, Chromolaena odorata*
156.	Genkwanin	*Odontites viscosus, Eupatorium capillifolium*
157.	Rhamnazin	*Rhamnus petiolaris*
158.	Mollic acid glucoside	*Combretum molle*
159.	Arjungenin	*Combretum punctatum, Rudgea viburnoides*
160.	Isoliquiritigenin	*Glycirrhiza glabra*
161.	Liquiritigenin	*Glycirrhiza glabra*
162.	Alpinetin	*Alpinia katsumadai*
163.	Cardamonin	*Alpinia katsumadai*
164.	N-trans-feruloyl 4′-O-methyldopamine	*Mirabilis jalapa*
165.	Alpha bisabolol	*Mirabilis jalapa*
166.	Juglone	*Talaromyces diversus, Carya alba*
167.	Tetradine	*Pachygone dasycarpa, Cyclea barbata*
168.	P-Coumaric acid	*Ficus septica, Visnea mocanera*
169.	Glycirrhetinic acid	*Glycirrhiza glabra*
170.	Licochalcone A	*Glycirrhiza glabra*
171.	Prenyllicoflavone A	*Glycirrhiza glabra*
172.	Glabrene	*Glycirrhiza glabra*
173.	Licocoumarin A	*Glycirrhiza glabra*
174.	18-beta-Glycyrrhetinic acid(enoxone)	*Glycirrhiza glabra*
175.	Liquiritin	*Glycirrhiza glabra*
176.	Kanzonol R	*Glycirrhiza glabra*
177.	alpha-Terpineol	*Glycirrhiza glabra*
178.	Shinpterocarpin	*Glycirrhiza glabra*
179.	1-Methoxyficifolinol	*Glycirrhiza glabra*
180.	Licoriphenone	*Glycirrhiza glabra*
181.	Licoarylcoumarin	*Glycirrhiza glabra*
182.	Furfuraldehyde	*Glycirrhiza glabra*
183.	Glisoflavone	*Glycirrhiza glabra*
184.	Isoangustone A	*Glycirrhiza glabra*
185.	2,3-Butanediol	*Glycirrhiza glabra*
186.	Semilicoisoflavone B	*Glycirrhiza glabra*
187.	Licopyranocoumarin	*Glycirrhiza glabra*
188.	Tetramethyl pyrazine	*Glycirrhiza glabra*
189.	Vasicine	*Adhatodavasica*
190.	Vasicol	*Adhatodavasica*
191.	Vasicinone	*Adhatodavasica*
192.	Vasicinolone	*Adhatodavasica*
193.	9,10-dihydro-2,5-dimethoxyphenanthrene-1,7-diol	*Eulophia nuda*
194.	9,10-dihydro-4-methoxyphenanthrene-2,7-diol	*Eulophia nuda*

195.	1,5-dimethoxyphenanthrene-2,7-diol	*Eulophia nuda*
196.	1,5,7,-trimethoxyphenanthrene-2,6-diol	*Eulophia nuda*
197.	5,7-dimethoxyphenanthrene-2,6-diol	*Eulophia nuda*
198.	5-p-trans-coumaroylquinic acid (I)	*Gynura bicolor*
199.	4-hydroxybenzoic acid	*Gynura bicolor*
200.	Kaempferol-3-O-rutinoside	*Gynura bicolor*
201.	3,5-dicaffeoylquinic acid	*Gynura bicolor*
202.	Chebulic acid	*Terminalia chebula*
203.	Gallic acid	*Terminalia chebula*
204.	Taraxerol	*Diospyros morrisiana, Liatris acidota*
205.	Quercitrin	*Xylopia emarginata, Lotus ucrainicus*
206.	Galanthimine(Galantamine)	*Pancratium trianthum, Lycoris sanguinea*
207.	Termilignan B	*Terminalia sericea*
208.	Leucopelargonidol(leucopelrgonidin)	*Albizia lebbeck, Areca catechu*
209.	Decarine	*Zanthoxylum beecheyanum, Aralia bipinnata*
210.	Arjunic acid	*Rhaphiolepisdeflexa, Terminalia elliptica*
211.	Piperine	*Piper longum, Piper nigrum*

Names of 210 phytocompounds and their sources

Table S2

Sl. No.	DOPE score	Model
1.	-43883.83984	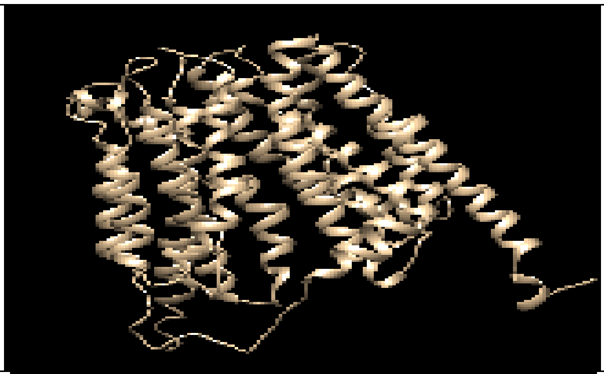
2.	-44076.47656	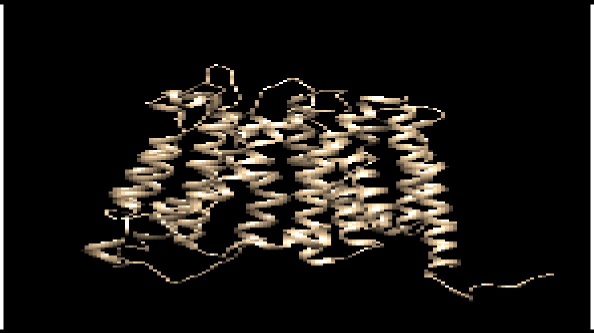
3.	-43790.26953	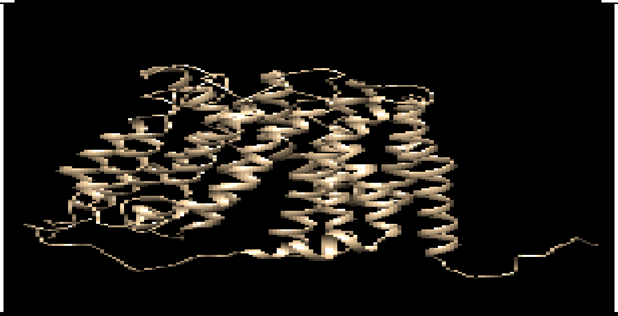
4.	-44255.55859	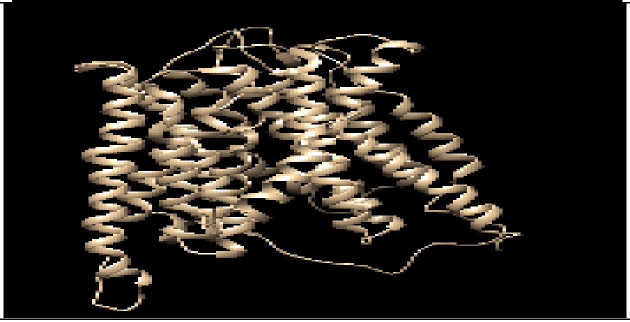

5.	-43891.86328	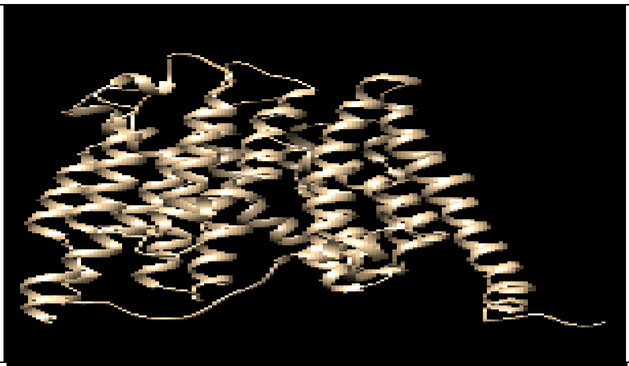
6.	-44188.64453	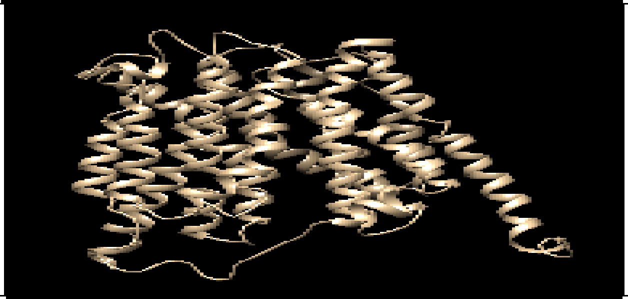
7.	-43956.07813	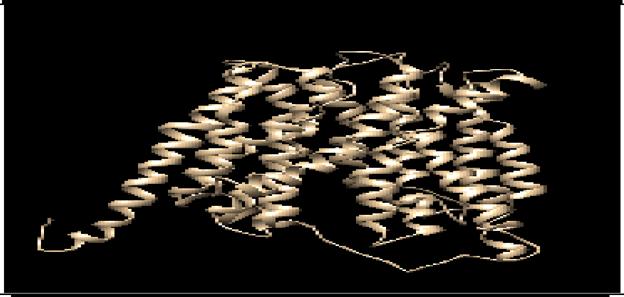
8.	-44463.44531	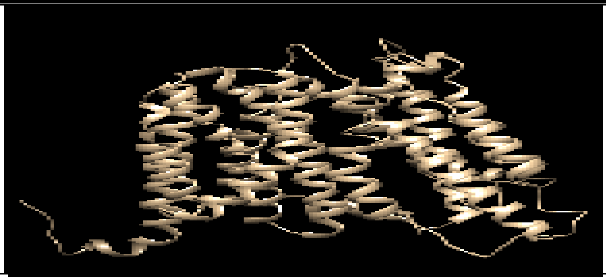
9.	**-44682.79688**	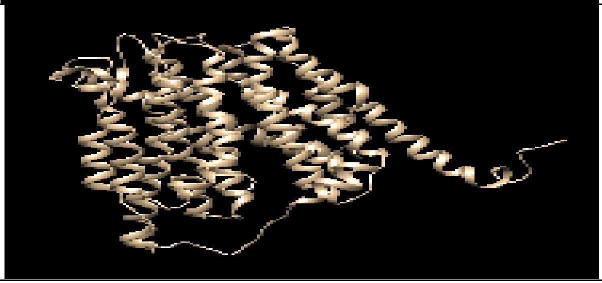

10.	-43829.83203		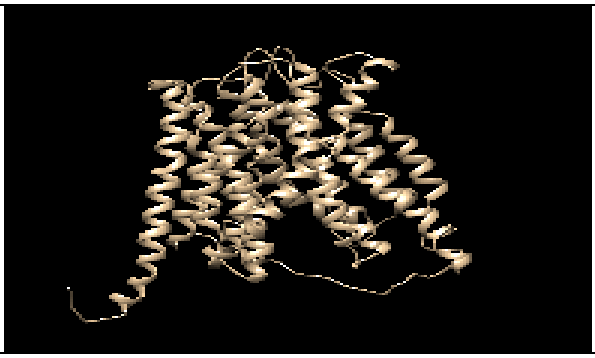	

DOPE scores along with predicted models

Fig. S3

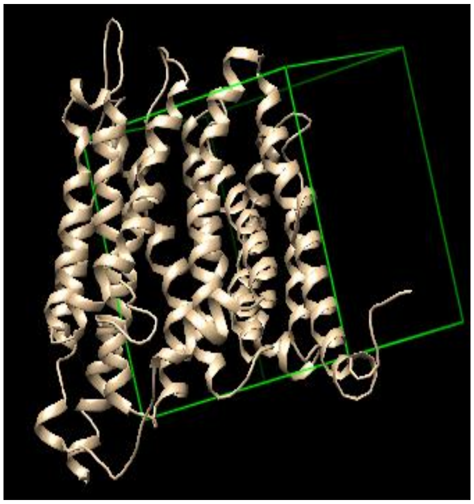

Grid box around the protein

Fig. S4

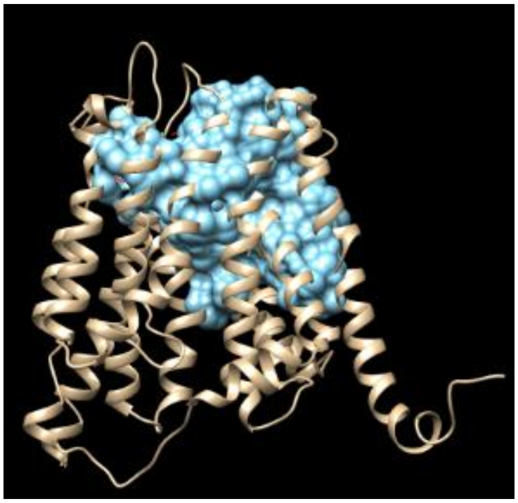

Active of the protein is shown

Fig.S5

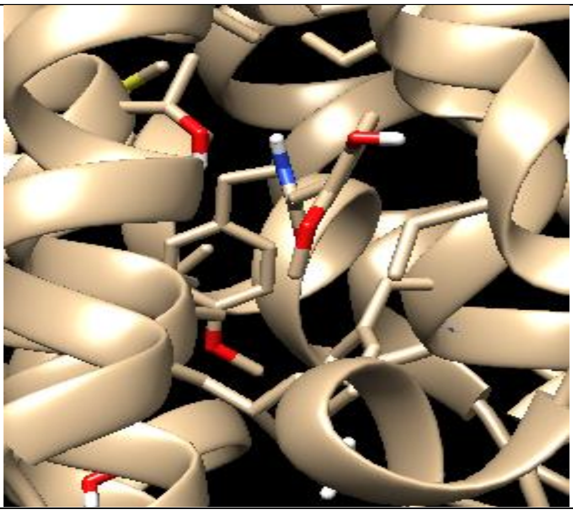	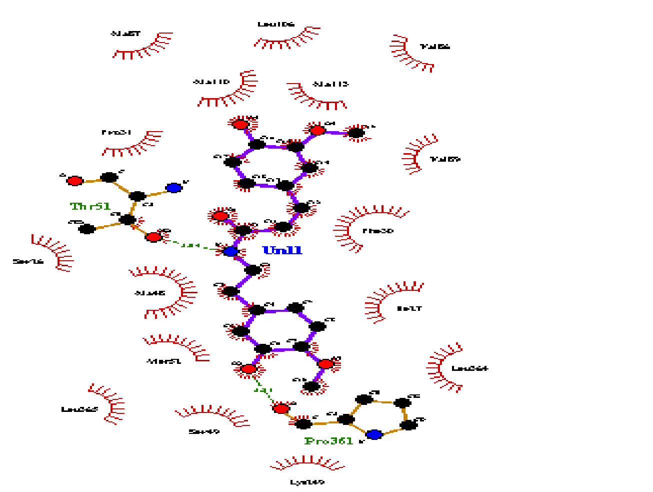
Fig.S5a i. Blind docked complex of N-transferuroyl-4’-O-methyldopamineand Rv1258c using UCSF Chimera software	Fig.S5a ii. Blind docked complex of abyssinone II and Rv1258c using LigPlot +.Green dotted lines show H bonds and Red curved lines show hydrophobicinteractions

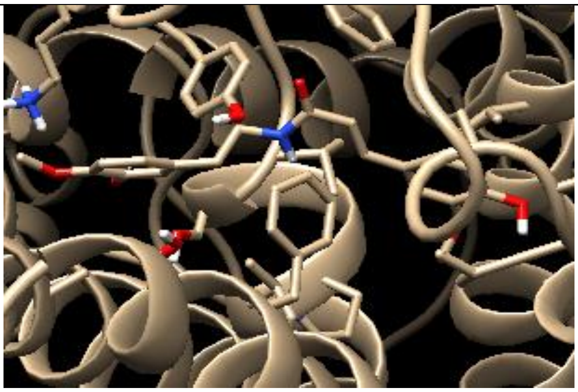

Fig. S5b i. Site specific docked complex of N-transferuroyl-4’-O-methyldopamineand Rv1258c using UCSF Chimera software

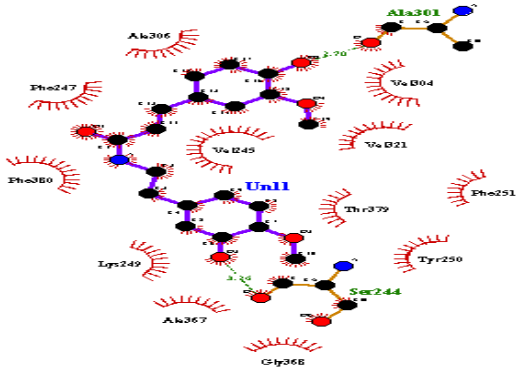

Fig. S5b ii. Site specific docked complex of of N-transferuroyl-4’-O-methyldopamine and Rv1258c using LigPlot +.Green dotted lines show H bonds and Red curved lines show hydrophobicinteractions

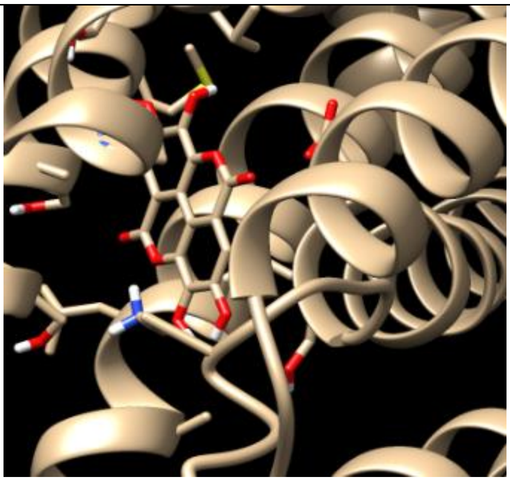

Fig S5c i. Blind docked complex of ellagic acid and Rv1258c using UCSF Chimera software

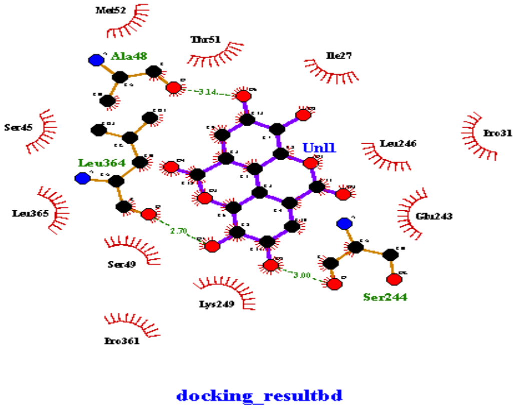

Fig S5c ii. Blind docked complex of ellagic acid and Rv1258c using LigPlot +.Green dotted lines show H bonds and Red curved lines show hydrophobic interactions

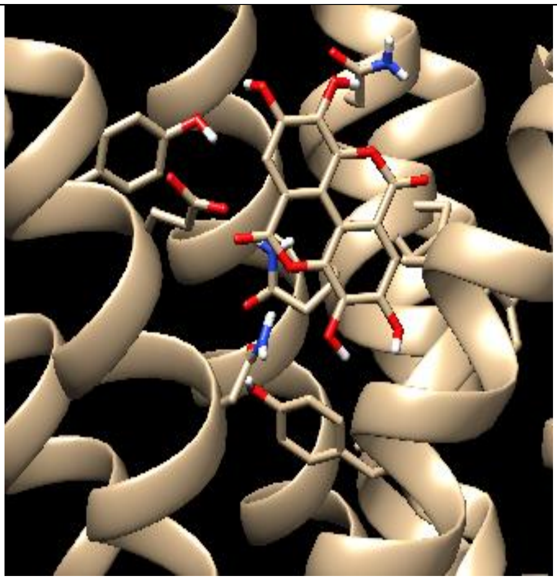

Fig.S5d i. Site specific docked complex of ellagic acid and Rv1258c using UCSF Chimera software

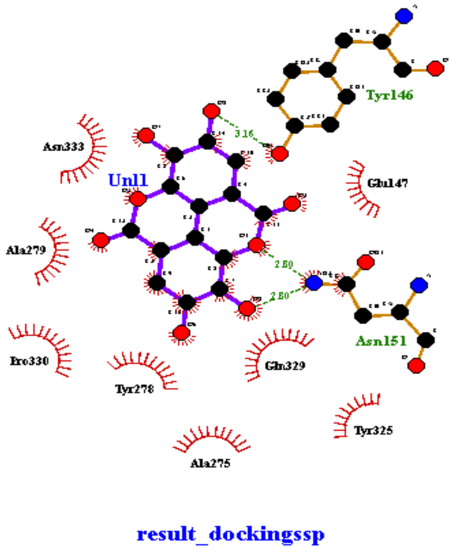

Fig.S5d ii. Site specific docked complex of ellagic acid and Rv1258c using LigPlot +.Green dotted lines show H bonds and Red curved lines show hydrophobic interactions

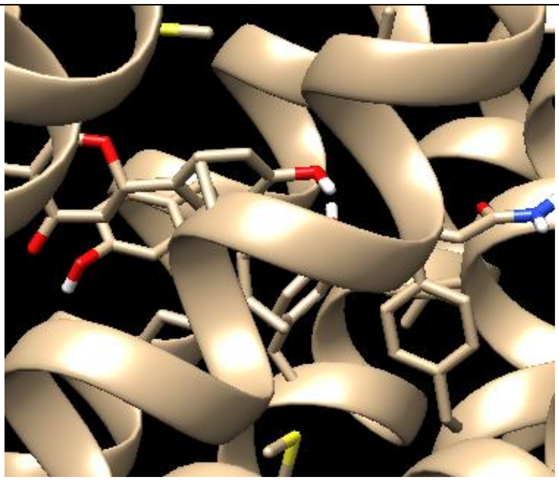

Fig.S5e i. Blind docked complex of abyssinone II and Rv1258c using UCSF Chimera software

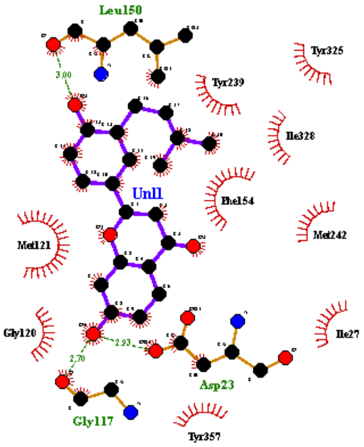

Fig. S5e ii. Blind docked complex of abyssinone II and Rv1258c using LigPlot +.Green dotted lines show H bonds and Red curved lines show hydrophobicinteractions

Fig.S3e

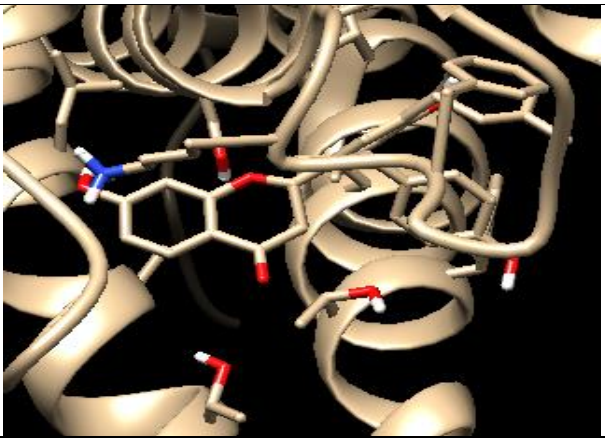

Fig.S5 f i. Site specific docked complex of abyssinone II and Rv1258c using UCSF Chimera software

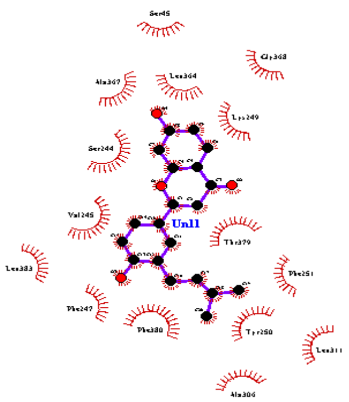

Fig.S5 f ii. Site specific docked complex of abyssinone II and Rv1258c using LigPlot +.Green dotted lines show H bonds and Red curved lines show hydrophobic interactions

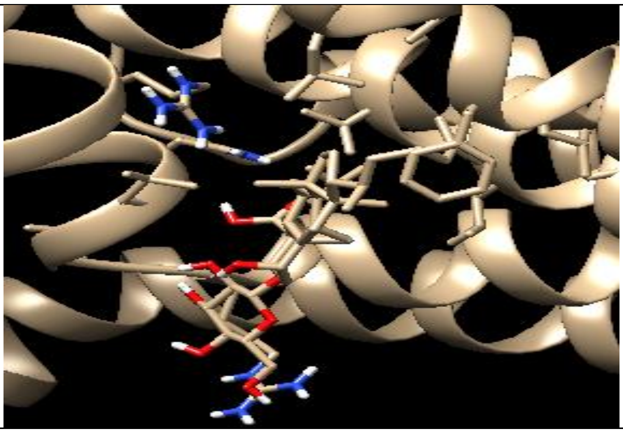

Fig S5g i. Blind docked complex of mollic acid glucoside nand Rv1258c using UCSF Chimera software

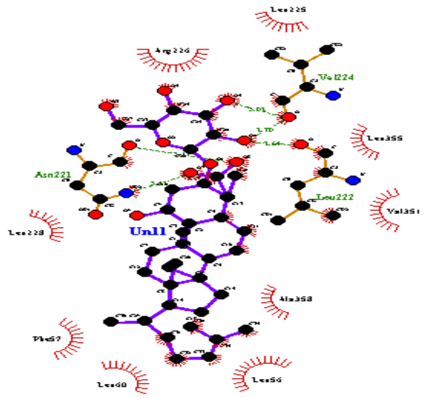

Fig S5g ii. Blind docked complex of mollic acid glucoside and Rv1258c using LigPlot +.Green dotted lines show H bonds and Red curved lines show hydrophobic interactions

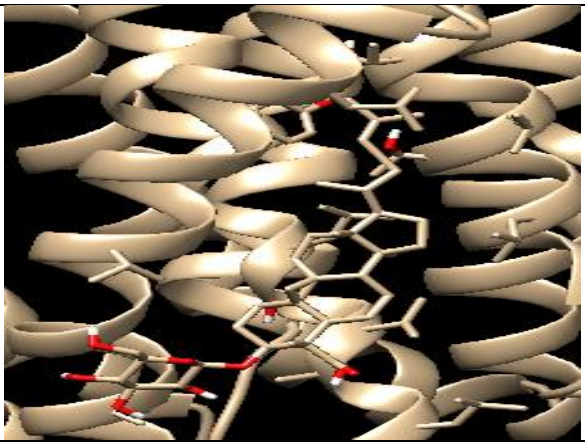

Fig.S5h i. Site specific docked complex of mollic acid glucoside nand Rv1258c using UCSF Chimera software

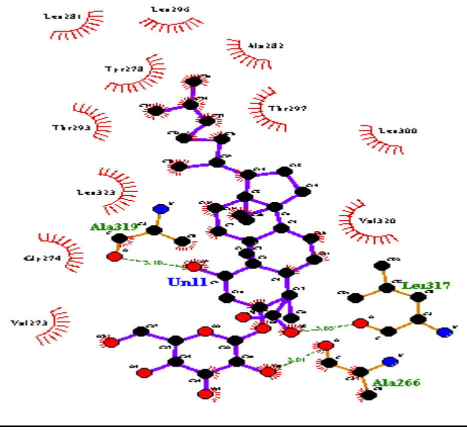

Fig.S5h ii. Site specific docked complex of mollic acid glucoside and Rv1258c using LigPlot +.Green dotted lines show H bonds and Red curved lines show hydrophobic interactions

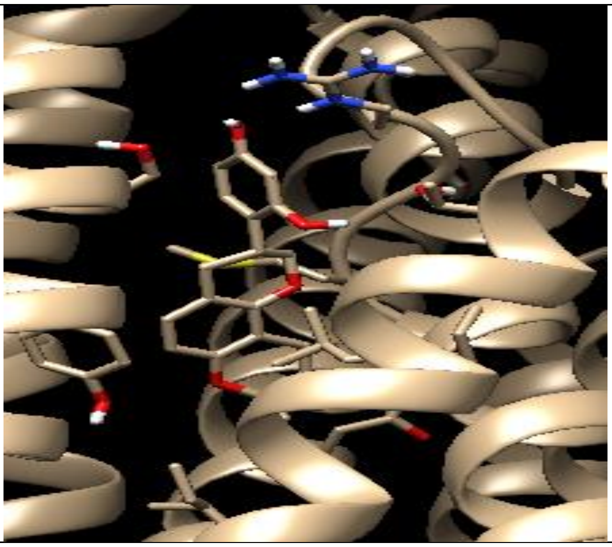

Fig.S5i i. Blind docked complex of glabridine and Rv1258c using UCSF Chimera software

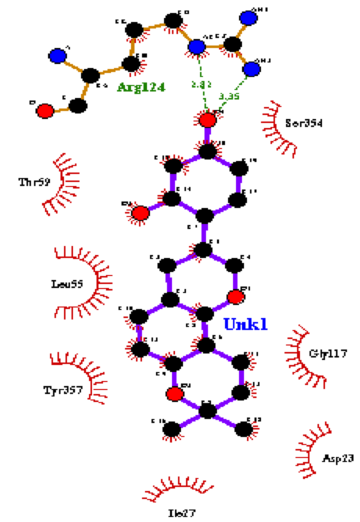

Fig.S5i ii. Blind docked complex of glabridine and Rv1258c using LigPlot +.Green dotted lines show H bonds and Red curved lines show hydrophobic interactions

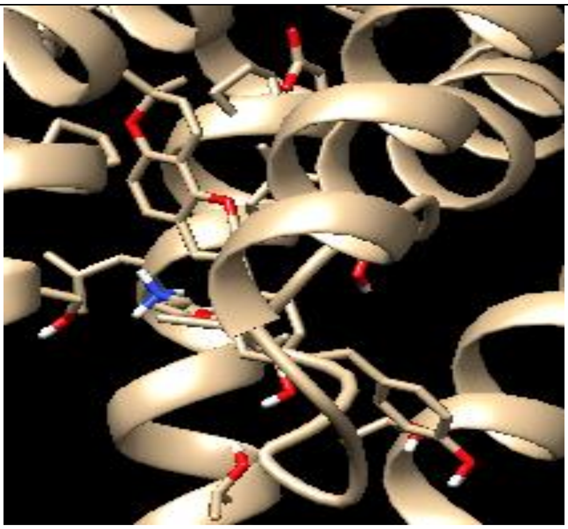

Fig.S5j i. Site specific docked complex of glabridine and Rv1258c using UCSF Chimera software

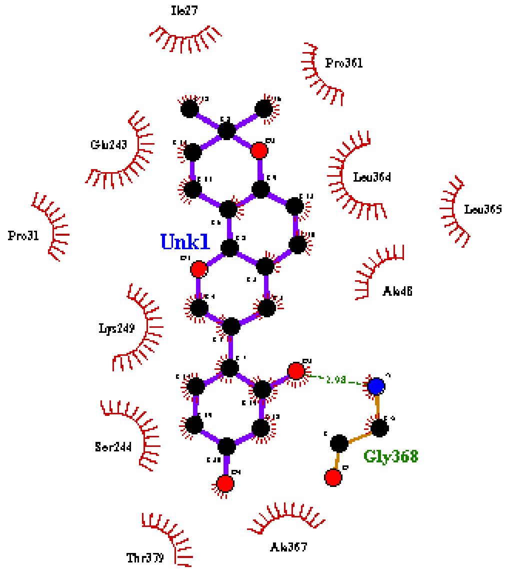

Fig.S5j ii. Site specific docked complex of glabridine and Rv1258c using LigPlot +.Green dotted lines show H bonds and Red curved lines show hydrophobic interactions

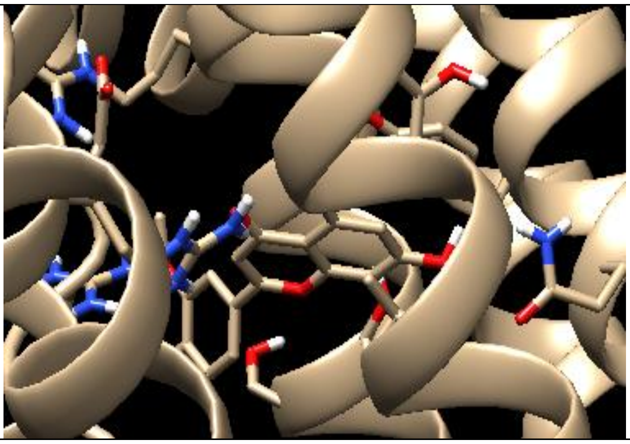

Fig.S5k i. Blind docked complex of chrysoeriol and Rv1258c using UCSF Chimera software

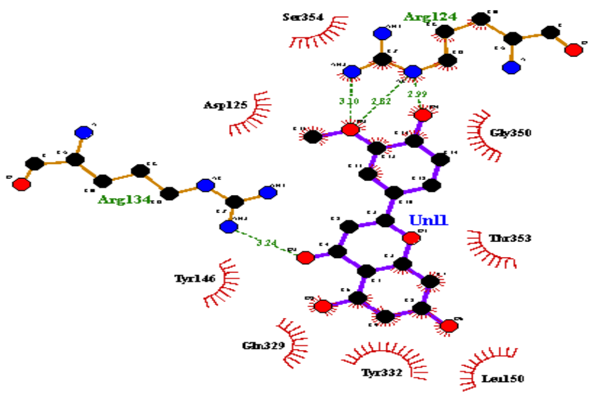

Fig.S5k ii. Blind docked complex of chrysoeriol and Rv1258c using LigPlot +.Green dotted lines show H bonds and Red curved lines show hydrophobic interactions

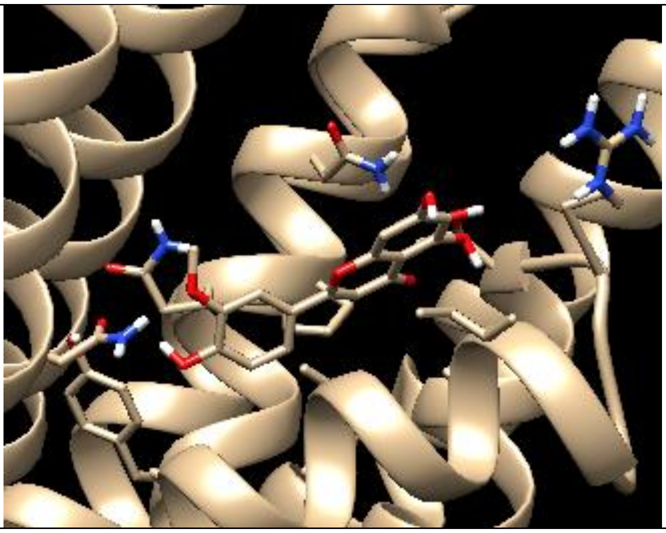

Fig.S5l i.Site specific docked complex of chrysoeriol and Rv1258c using UCSF Chimera software

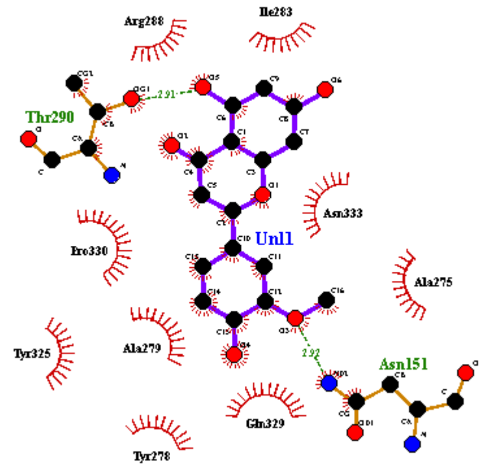

Fig.S5l ii. Site specific docked complex of chrysoeriol and Rv1258c using LigPlot +.Green dotted lines show H bonds and Red curved lines show hydrophobic interactions

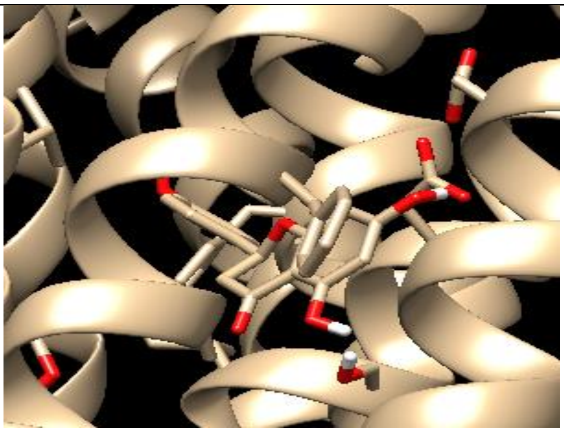

Fig.S53m i. Blind docked complex of naringenin and Rv1258c using UCSF Chimera software

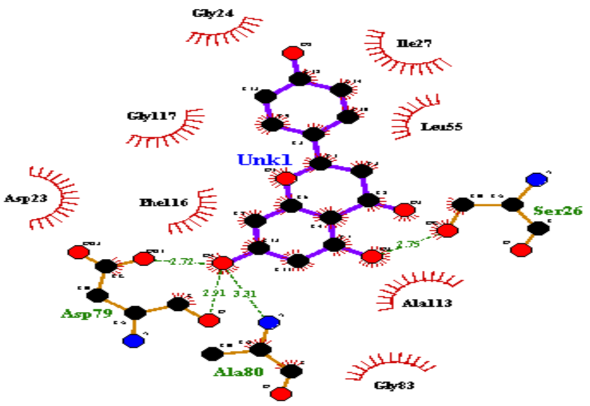

Fig.S5m ii. Blind docked complex of naringenin and Rv1258c using LigPlot +.Green dotted lines show H bonds

	and Red curved lines show hydrophobic interactions
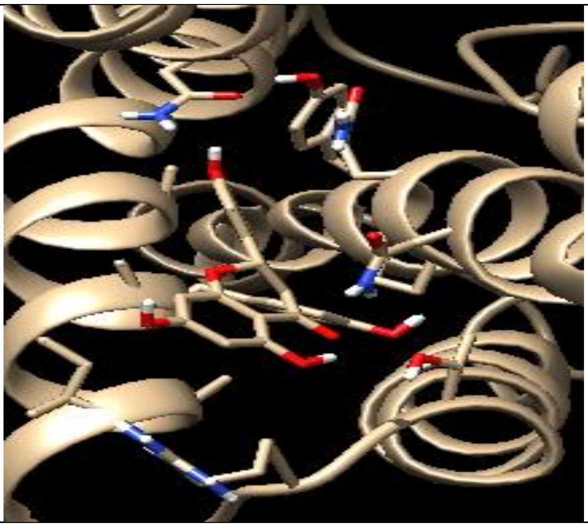	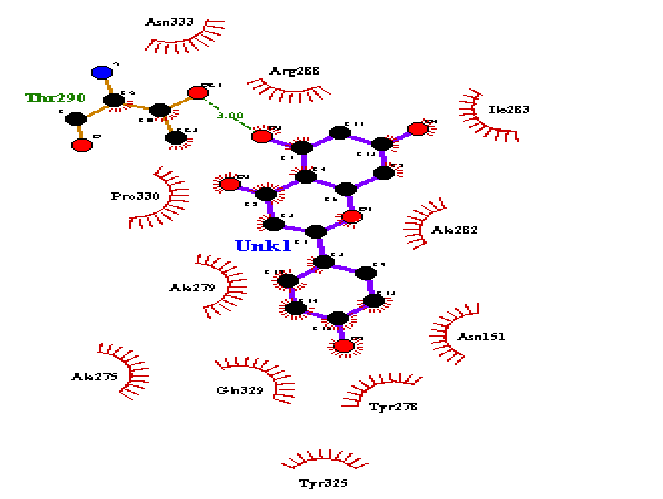
Fig.S5n i. Site specific docked complex of naringenin and Rv1258c using UCSF Chimera software	Fig.S5n ii. Site specific docked complex of naringenin and Rv1258c using LigPlot +.Green dotted lines show H bonds and Red curved lines show hydrophobic interactions
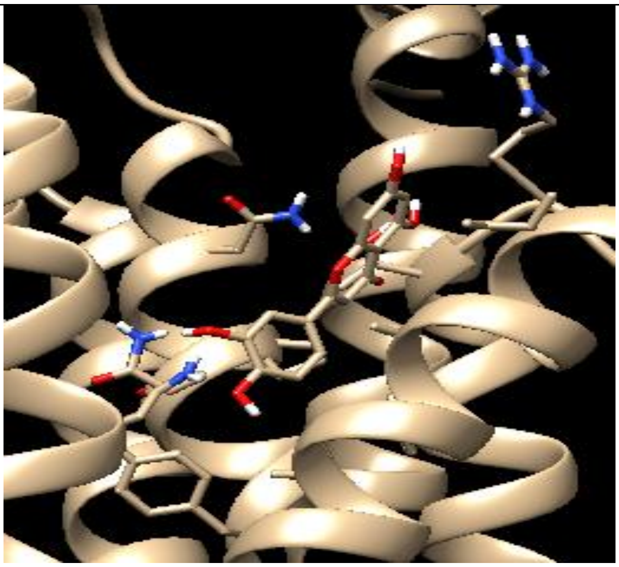	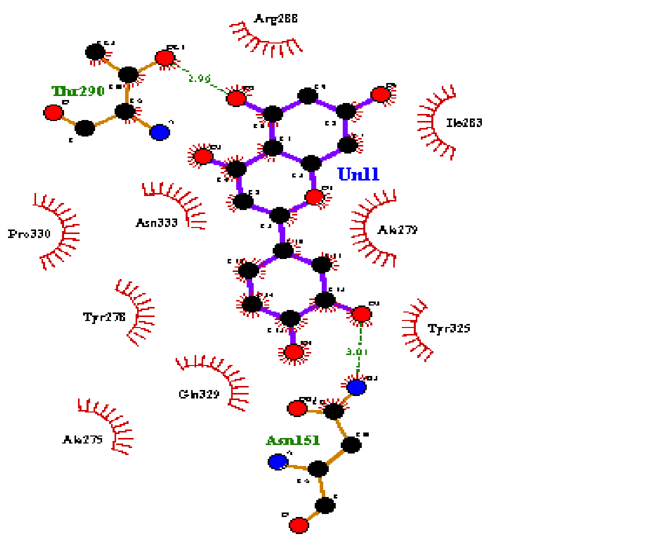
Fig.S5o i. Blind docked complex of luteolin and Rv1258c using UCSF Chimera software	Fig.S5o ii. Blind docked complex of luteolin and Rv1258c using LigPlot +.Green dotted lines show H bonds and Red curved lines show hydrophobic interactions

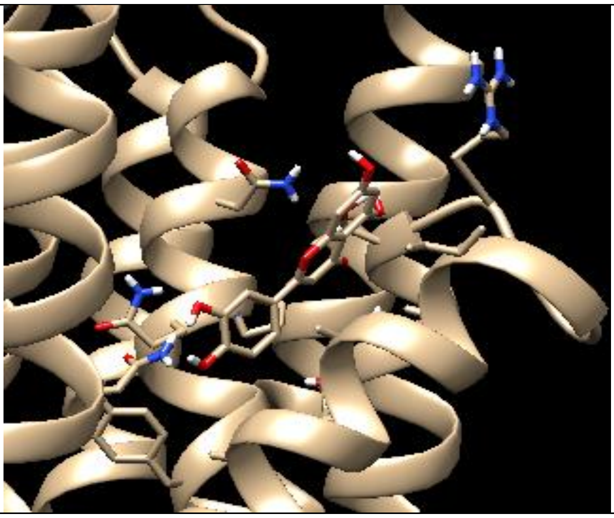	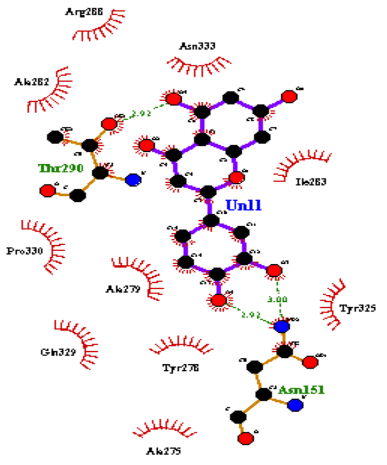
Fig.S5p i. Site specific docked complex of luteolin and Rv1258c using UCSF Chimera software	Fig.S5p ii. Site specific docked complex of luteolin and Rv1258c using LigPlot +.Green dotted lines show H bonds and Red curved lines show hydrophobic interactions
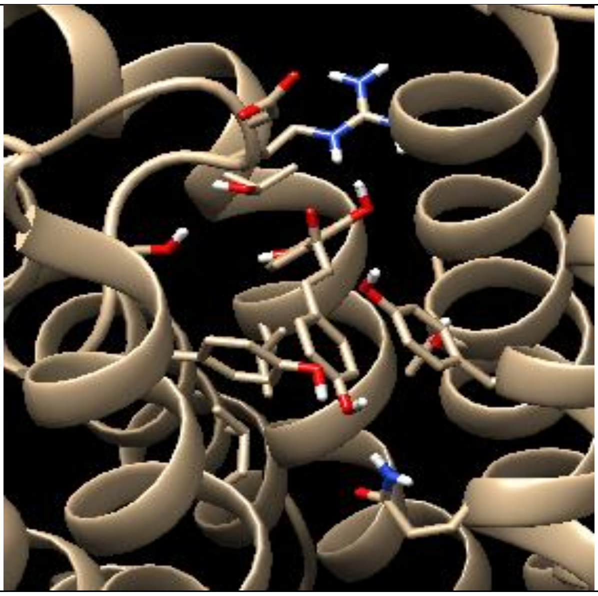	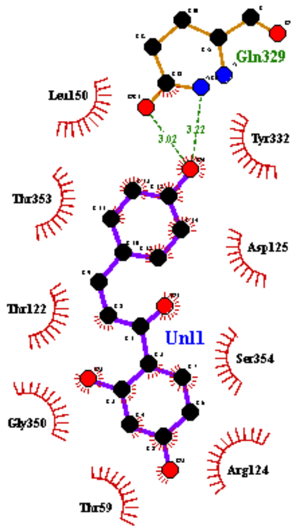
Fig.S5q i. Blind docked complex of isoliuiritigenin and Rv1258c using UCSF Chimera software	Fig.S5q ii. Blind docked complex of isoliquiritigenin and Rv1258c using LigPlot +.Green dotted lines show H bonds and Red curved lines show hydrophobic interactions

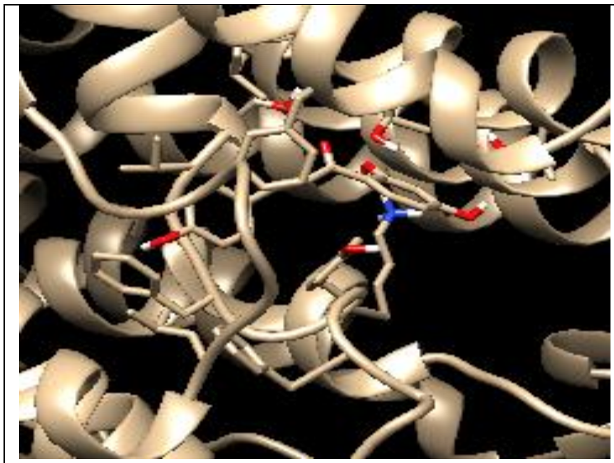

Fig.S5r i. Specific docked complex of isoliuiritigenin and Rv1258c using UCSF Chimera software

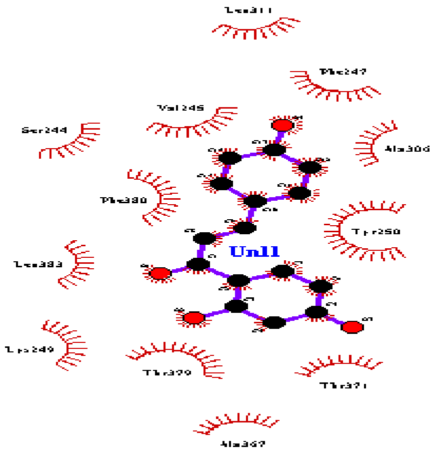

Fig.S5r ii. Site specific docked complex of isoliquiritigenin and Rv1258c using LigPlot +.Green dotted lines show H bonds and Red curved lines show hydrophobic interactions

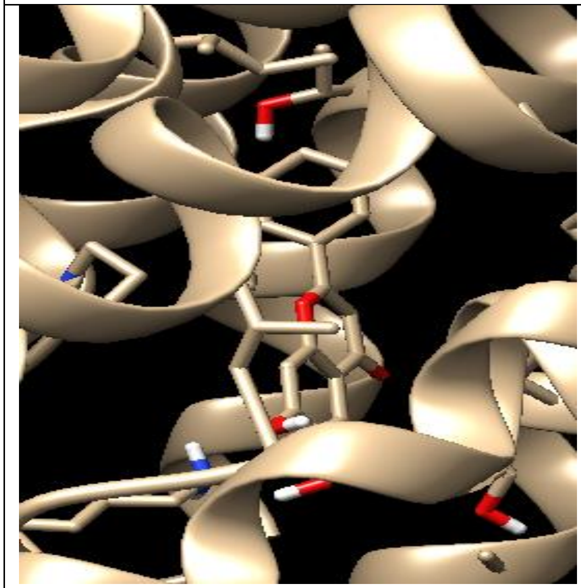

Fig.S5s i. Blind docked complex of baicalein and Rv1258c using UCSF Chimera software

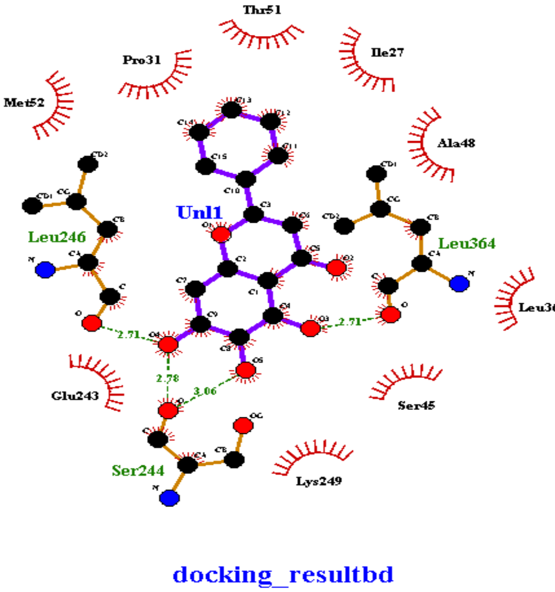

Fig.S5s ii. Blind docked complex of baicalein and Rv1258c using LigPlot +.Green dotted lines show H bonds and Red curved lines show hydrophobic interactions

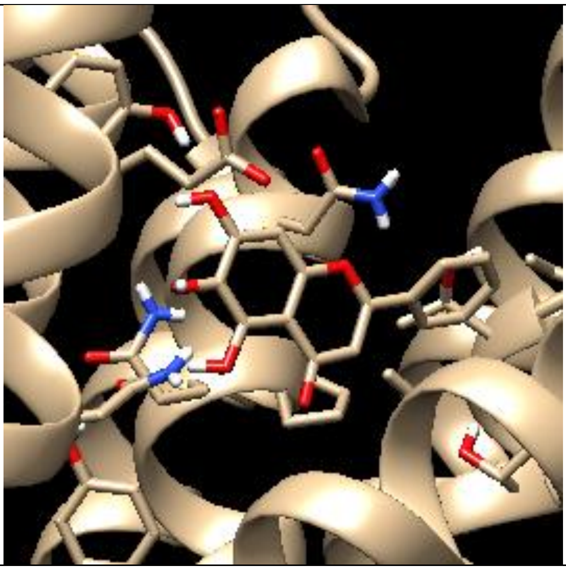

Fig.S5t i. Site specific docked complex of baicalein and Rv1258c using UCSF Chimera software

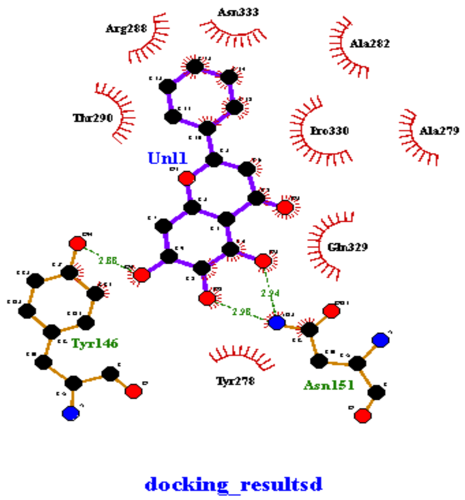

Fig.S5t ii. Site specific docked complex of baicalein and Rv1258c using LigPlot +.Green dotted lines show H bonds and Red curved lines show hydrophobic interactions

The images of the complexes formed of 10 best ligands with Rv1258c during blind and site specific docking and also the images of the complexes in LigPlot+ v.2.2.5 are shown in Fig. S5a i to S5t ii
